# A deep-learning framework reveals whole-body perturbations at cell level

**DOI:** 10.1038/s41586-026-10535-2

**Published:** 2026-05-20

**Authors:** Doris Kaltenecker, Izabela Horvath, Rami Al-Maskari, Ying Chen, Zeynep Ilgin Kolabas, Luciano Hoeher, Mihail Todorov, David-Paul Minde, Saketh Kapoor, Sena Gül Turhan, Louis B. Kuemmerle, Hanno Steinke, Tim Wohlgemuth, Mayar Ali, Florian Kofler, Pauline Morigny, Julia Geppert, Denise Jeridi, Bastian Wittmann, Jie Luo, Suprosanna Shit, Carolina Cigankova, Victor Miro Kolenic, Nilsu Gür, Eren Aydeniz, Alara Yücecan, Melissa Ertürk, Laurent H. A. Simons, Chenchen Pan, Marie Piraud, Daniel Rueckert, Maria Rohm, Farida Hellal, Markus Elsner, Harsharan Singh Bhatia, Ingo Bechmann, Bjoern H. Menze, Stephan Herzig, Johannes Christian Paetzold, Mauricio Berriel Diaz, Ali Ertürk

**Affiliations:** 1 Institute for Diabetes and Cancer (IDC), Helmholtz Munich, German Research Center for Environmental Health, Neuherberg, Germany; 2 Joint Heidelberg-IDC Translational Diabetes Program, Heidelberg University Hospital, Heidelberg, Germany; 3 German Center for Diabetes Research (DZD), Neuherberg, Germany; 4 Institute for Intelligent Biotechnologies, Helmholtz Munich, German Research Center for Environmental Health, Neuherberg, Germany; 5 TUM School of Computation, Information and Technology (CIT), Technical University of Munich, Munich, Germany; 6 Institute for Stroke and Dementia Research, Klinikum der Universität München, Ludwig-Maximilians-Universität LMU, Munich, Germany; 7 Munich Medical Research School (MMRS), Munich, Germany; 8 Institute of Computational Biology, Helmholtz Munich, Neuherberg, Germany; 9 School of Life Sciences Weihenstephan, Technical University of Munich, Freising, Germany; 10 Institute of Anatomy, University of Leipzig, Leipzig, Germany; 11 Helmholtz AI, Helmholtz Munich, Neuherberg, Germany; 12 Department for Quantitative Biomedicine, University of Zurich, Zurich, Switzerland; 13 AI for Image-Guided Diagnosis and Therapy, TUM School of Medicine and Health, Technical University of Munich, Munich, Germany; 14 Hertie Institute for AI in Brain Health, University of Tübingen, Tübingen, Germany; 15 Department of Industrial and Molecular Pharmaceutics, Purdue University, West Lafayette, IN USA; 16 Purdue University Institute for Cancer Research, West Lafayette, IN USA; 17 Interdisciplinary Research Center on Biology and Chemistry, Shanghai Institute of Organic Chemistry, Chinese Academy of Sciences, Shanghai, China; 18 Department of Computing, Imperial College London, London, United Kingdom; 19 Chair for AI in Healthcare and Medicine, Technical University of Munich (TUM) and TUM University Hospital, Munich, Germany; 20 Chair Molecular Metabolic Control, TU, Munich, Germany; 21 Department of Radiology, Weill Cornell Medicine, New York, NY USA; 22 Cornell Tech, New York, NY USA; 23 School of Medicine, Koç University, Istanbul, Turkey; 24 Munich Cluster for Systems Neurology (SyNergy), Munich, Germany; 25 Deep Piction, Munich, Germany

**Keywords:** Obesity, Machine learning, Systems analysis

## Abstract

Many diseases, including obesity, have systemic effects that perturb multiple organ systems throughout the body^1,2^. However, tools for comprehensive, high-resolution analysis of disease-associated changes at the whole-body scale have been lacking. Here we developed MouseMapper, a suite of foundation-model-based deep-learning algorithms enabling multi-system analysis of disease across the entire mouse body. MouseMapper enables whole-body quantitative analysis of nerves and immune cells, resolving fine axonal branches and immune-cell clusters while automatically segmenting 31 organs and tissues. We used MouseMapper to study diet-induced obesity, and identified structural alterations of the infraorbital branch of the trigeminal ganglia. This structural impairment in infraorbital nerves was associated with functional sensory deficits in whisker sensing. Furthermore, we identified proteomic changes in the trigeminal ganglion affecting axon remodelling and complement pathways both in mice and humans. MouseMapper also generated detailed three-dimensional inflammation maps by characterizing immune cell cluster compositions across tissues. The MouseMapper framework demonstrates robust generalizability across different imaging resolutions and datasets. Our study provides a powerful, scalable approach for identifying and quantifying systemic pathologies, bridging molecular insights from animal models to human conditions.

## Abstract (web summary)

An analysis of diet-induced obesity using MouseMapper—a suite of foundation-model-based deep-learning algorithms—identifies structural alterations of the infraorbital branch of the trigeminal ganglia.

## Main

Many diseases, including lifestyle-induced conditions such as obesity, have far-reaching effects that impact multiple organ systems throughout the body. These systemic effects underscore the interconnected nature of body physiology and the need for holistic approaches to understanding pathological changes. However, tools to study cellular and molecular perturbations at the whole-body scale have been lacking, limiting our ability to understand their broad impacts.

Advanced tissue-clearing methods combined with fluorescence microscopy have enabled the visualization of large samples, including entire mouse bodies and big human tissue specimens at the single-cell resolution^1–5^. However, the lack of suitable image analysis tools to quantify cellular and elongated structures such as nerves, tissues and organs at the whole-body scale has been a major bottleneck for identifying structural alterations in response to disease. Although several approaches for image analysis of light-sheet fluorescence microscopy (LSFM) datasets have been introduced in recent years^6–10^, these methods are typically limited to selected organs and do not provide integrated whole-body quantification of elongated structures. Furthermore, developing algorithms that robustly generalize across different imaging resolutions and labelling strategies has remained a significant challenge. Such tools would allow defining regions for further molecular characterization to reveal the mechanisms governing the systemic effects of diseases^11^.

Obesity is associated with chronic low-grade inflammation and a plethora of metabolic dysfunctions such as insulin resistance, impaired glucose tolerance and hypertension. Obesity increases the risk of developing secondary comorbidities, including type 2 diabetes, peripheral neuropathies, cardiovascular diseases, stroke and numerous cancer types^12,13^, underscoring the systemic effects of excess body fat and the need for holistic characterizations of the underlying structural and cellular changes.

Here we developed MouseMapper—a deep-learning framework built on a foundation model (VesselFM)^14^ for three-dimensional (3D) image analysis to segment and analyse whole-body images of the nervous and immune systems and select regions of interest for subsequent molecular analysis. MouseMapper has three modules for (1) quantitative analysis of peripheral nerve networks; (2) the segmentation and quantification of immune cells; and (3) artificial intelligence (AI)-based mapping of the segmented structures to 31 organs and tissues across entire mouse bodies. The framework’s design demonstrates robust generalizability across different imaging resolutions and antibody-labelled datasets without retraining. Using MouseMapper, we identified structural alterations in nerve and immune cell networks with high spatial resolution. Among others, we identified structural changes of the infraorbital branch of the trigeminal nerve in obese mice, which was associated with functional sensory deficits in whisker sensing and proteomic alterations related to axon degeneration and remodelling—a molecular signature that we found to be conserved in trigeminal ganglia of obese humans.

## Whole-body nerve and immune visualization

**Fig. 1:**
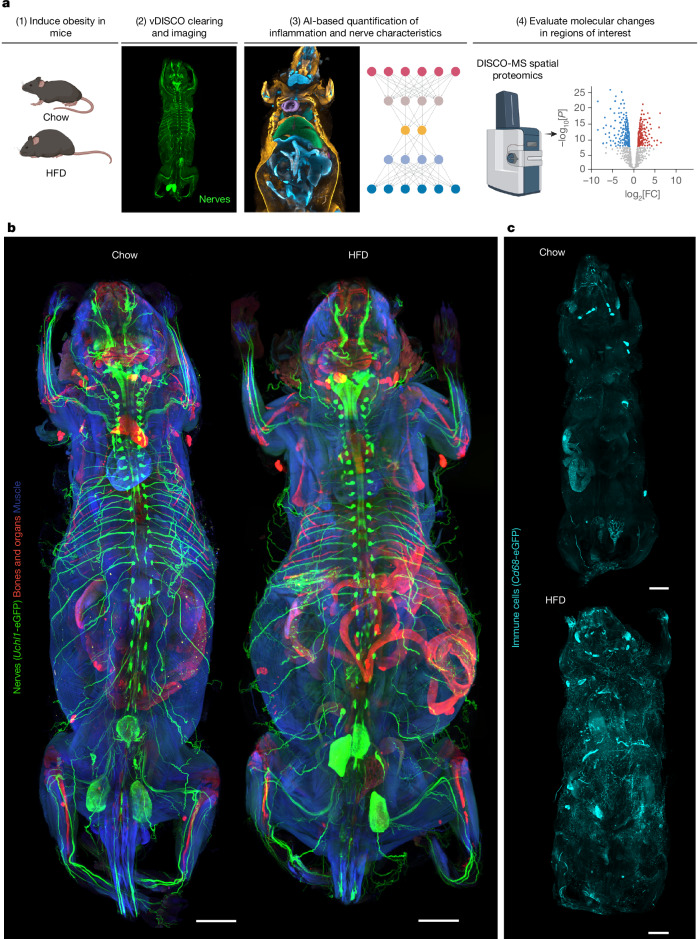
Direct visualization of nerves and macrophages in obesity at the whole-body scale. **a**, Workflow to study obesity-induced changes at the whole-body scale. The diagram was created using BioRender; Kaltenecker, D. https://biorender.com/t34651c (2026).** b**,**c**, Representative 3D reconstructions of vDISCO-cleared and imaged chow-fed and HFD-fed mice, showing *Uchl1*-eGFP^+^ peripheral nerves (**b**) (*n* = 4 chow and *n* = 5 HFD-fed mice) and *Cd68-*eGFP^+^ immune cells (**c**) (*n* = 5 chow, *n* = 5 HFD-fed mice). Scale bars, 5,000 μm (**b**,**c**).

**Extended Data Fig. 1:**
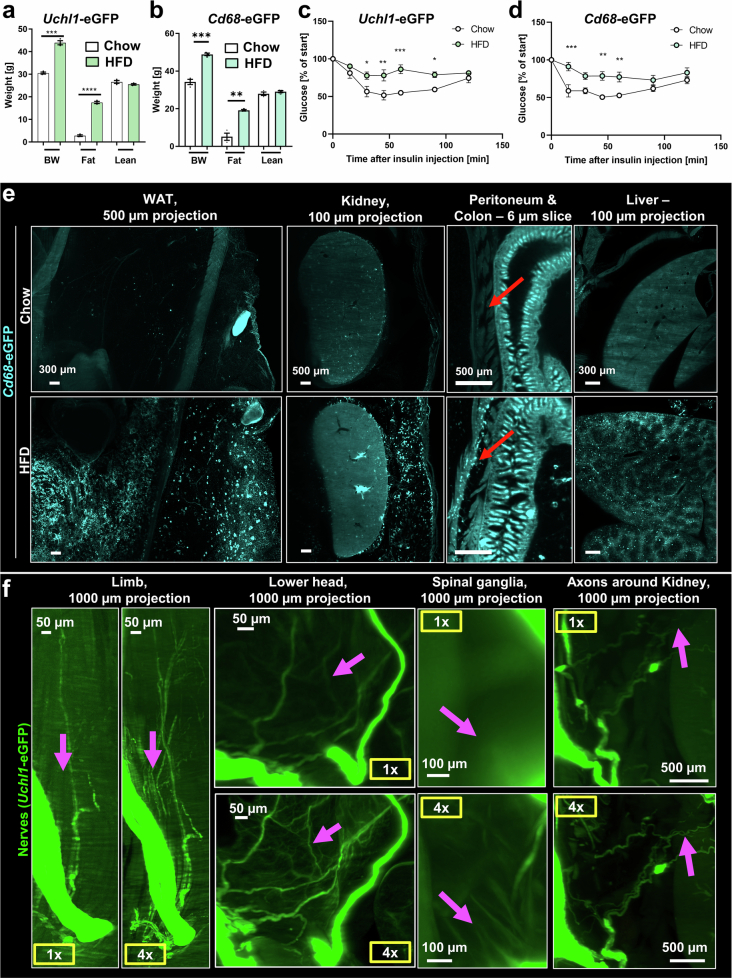
HFD-feeding associated with increased body weight and macrophage infiltration. **a**,**b**, Body composition analysis using EchoMRI of *Uchl1*, (n = 3/group, two-tailed t-test, p-values: 0.0002 (BW), <0.0001 (Fat)) and *Cd68*-eGFP mice (n = 3/group, two-tailed t-test, p-values: 0.0007 (BW), 0.0019 (Fat)). BW: Body weight. **c**,**d** Insulin tolerance test of *Uchl1* (n = 4 chow, n = 5 HFD, two way ANOVA with Sídak’s multiple comparisons test, p-values: two-tailed t-test, p-values: 0.021 (30 min), 0.0022 (45 min), 0.0002 (60 min), 0.0389 (90 min)) and *Cd68*-eGFP mice (n = 5 chow, n = 5 HFD, two way ANOVA with Sídak’s multiple comparisons test, p-values: two-tailed t-test, p-values: 0.0002 (15 min), 0.0013 (45 min), 0.0069 (60 min)). *p < 0.05, **p < 0.01, ***p < 0.001, ****p < 0.0001, data are represented as mean ± standard error. **e**, Representative images (1x objective, 0.1NA) of indicated areas in chow and HFD-fed mice showing *Cd68*-eGFP+ cells in cyan (n = 5 chow, n = 5 HFD). **f**, Representative light-sheet images of indicated areas that were acquired using a 1.1x (0.1 Numerical aperture (NA) and a 4x objective (0.35 NA) (n = 4 chow, n = 5 HFD). Source data

**Extended Data Fig. 2:**
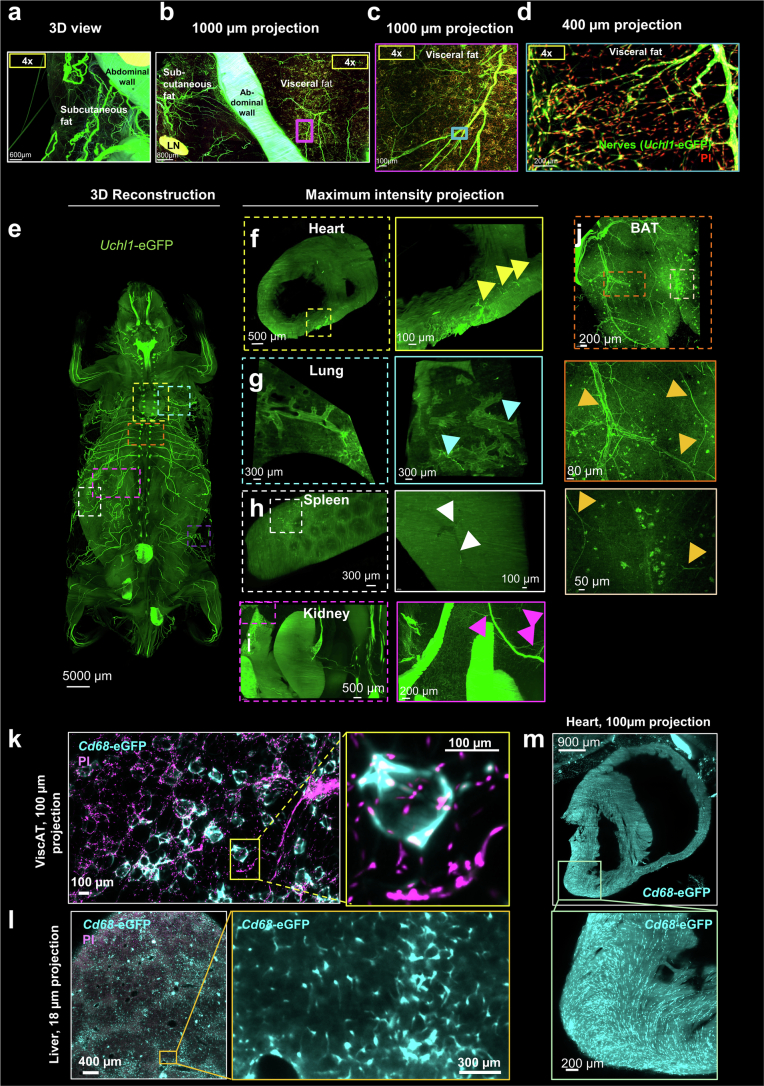
4x imaging allows to see finer structural details. **a-d**, Representative 3D reconstruction of the lower belly area (**a**) and projections of adipose tissue at different zoom in levels of a HFD-fed *Uchl1*-eGFP animal using the 4x objective (0.35 NA) to showcase expression of *Uchl1*-eGFP+ nerves shown in green in all panels and PI showing the nucleus in red (**c**,**d**). LN: Lymph node. **e**, Representative 3D reconstruction of a HFD-fed *Uchl1*-eGFP animal to showcase expression of *Uchl1*-eGFP+ nerves shown in green in panels e-i. **f-i**, Zoomed in views of dashed regions in (**f**) showing the heart demonstrating the nerves in the heart with yellow arrows, lungs in (**g**) nerves with blue arrows, spleen in (**h**), nerves with white arrows, abdominal cavity with the kidneys and a part of the spleen in (**i**), nerves in the visceral fat are shown here with pink arrows, brown adipose tissue (BAT) in (**j**) different zoomed in regions show the innervation with orange arrows. The thickness of the MIPs are as follows: 500 µm in (f), 100 µm in (g), 230 µm for left and 500 µm for right panel in (h), 1500 µm in (i), 600 µm in (j). **k-m**, Representative images of tissues from obese *Cd68*–eGFP mice. Dashed boxes indicate regions shown at higher zoom-ins in the adjacent Images in Z-projections. Images were acquired with a 4x objective (0.35NA) and are shown using different brightness and contrast settings in different organs (n = 5 chow, n = 5 HFD).

**Extended Data Fig. 3:**
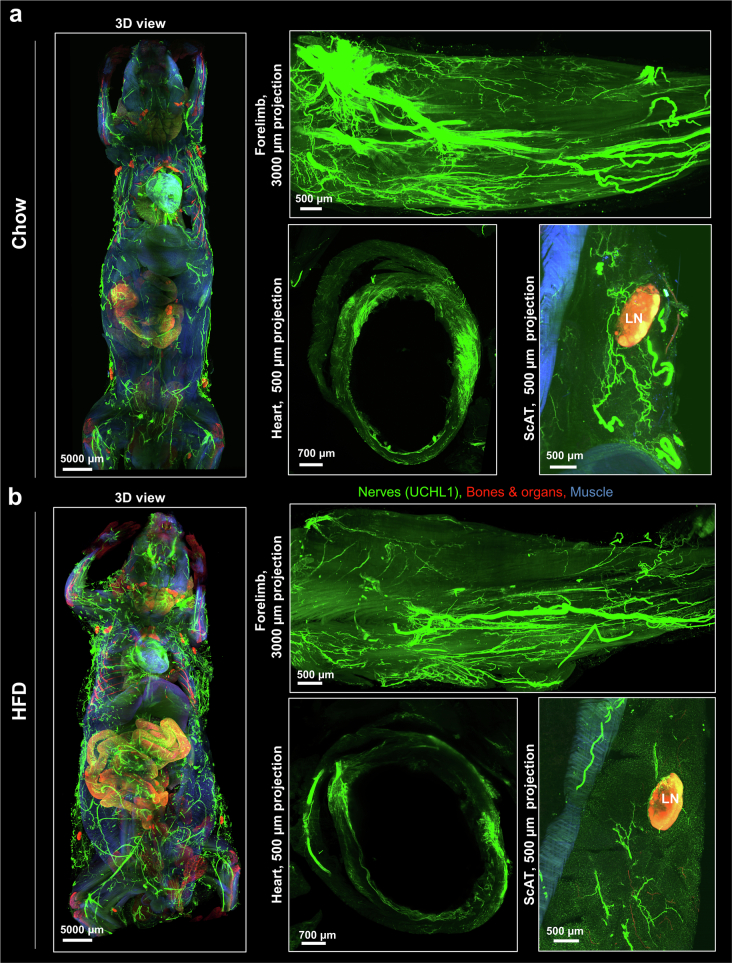
Visualization of the WildDISCO antibody labelling in obese mice. **a**,**b**, 3D representation of ventral light-sheet fluorescence microscopy scan of wildtype mice that were antibody labelled with the pan-nerve marker UCHL1 in chow (a) and HFD-fed (b) mice (n = 3/group).

**Extended Data Fig. 4:**
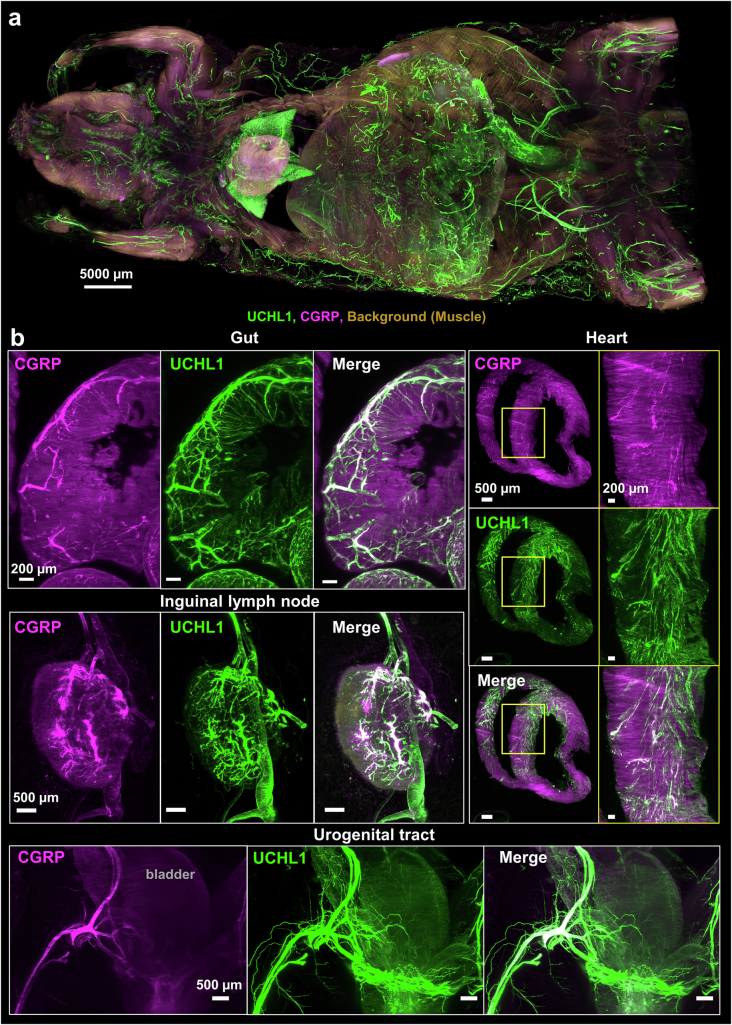
Visualization of the WildDISCO double labelling. **a**,**b**, 3D representation of ventral light-sheet fluorescence microscopy scan of a wildtype mouse (n=1) that was antibody labelled with the pan-nerve marker UCHL1 and a sensory nerve marker (CGRP). Approximately 64% of UCHL1^+^ nerves were positive for CGRP.

In this study we aimed to develop a comprehensive toolset for studying disease-induced whole-body changes, specifically for the study of obesity. To this end, we subjected mice that express eGFP under the peripheral nerve marker *Uchl1* (also known as PGP9.5) promoter (*Uchl1-*eGFP mice) or the monocyte/macrophage marker *Cd68* promoter (*Cd68-*eGFP mice) to high-fat diet (HFD) feeding for 16–18 weeks. This led to significantly increased body weights compared with the chow-fed controls, mainly due to increased adipose tissue, while lean mass remained similar (Fig. 1a and Extended Data Fig. 1a,b). HFD feeding was associated with impaired insulin response, demonstrating successful induction of metabolic dysfunction in the reporter mice (Extended Data Fig. 1c,d). vDISCO clearing and LSFM of transparent mouse bodies (×1.1 objective, 0.1 numerical aperture (NA)) enabled whole-body visualization of the peripheral nervous system (Fig. 1b and Supplementary Video 1) and *Cd68-*eGFP^+^ immune cells (Fig. 1c and Supplementary Video 2) in 3D in lean mice and large obese mouse bodies. For example, in *Uchl1*-eGFP mice, we could clearly trace nerve bundles over lengths of several centimetres, including their paths from the dorsal root ganglia (DRG) into the subcutaneous adipose tissue (ScAT) depot (Fig. 1b and Supplementary Videos 3 and 4). In obese *Cd68*-eGFP mice, an increase in *Cd68-*eGFP^+^ cell infiltration was apparent throughout the mouse body compared with in the chow-fed controls, with the most prominent accumulations in the liver and visceral adipose tissue (ViscAT), including epididymal, mesenteric, perirenal and cardiac ectopic adipose tissue (Fig. 1c, Extended Data Fig. 1e and Supplementary Videos 5 and 6). Higher-resolution LSFM acquisitions (×4 objective, 0.35 NA) provided enhanced structural detail compared with images acquired with the ×1.1 objective (Extended Data Fig. 1f). This increase in NA and spatial resolution enabled the visualization of thinner axonal structures, particularly in adipose tissue (Extended Data Fig. 2a–d). *Uchl1*-eGFP^+^ nerves were also visible in internal organs such as heart, liver, spleen and kidneys as well as in brown fat (Extended Data Fig. 2e–j). We also observed discrete* Cd68-*eGFP^+^ immune cells within the adipose tissue, liver and heart (Extended Data Fig. 2k–m and Supplementary Video 7), highlighting the ability of ×4 imaging to resolve more detailed neural and immune features in intact organs. In addition to reporter mice, we also imaged whole-mouse bodies labelled with antibodies against the pan-peripheral nerve marker UCHL1. Although this approach produced high-quality staining in selected regions, including the heart, limbs and ScAT, the labelling appeared incomplete in larger HFD-fed mouse bodies, limiting the ability to obtain consistent whole-body nerve maps (Extended Data Fig. 3a,b). We also performed double labelling with the sensory nerve marker CGRP alongside UCHL1 (Extended Data Fig. 4a). Although this revealed CGRP^+^ and CGRP^−^ subsets of axonal projections, the antibody staining did not produce uniform coverage throughout large HFD-fed specimens (Extended Data Fig. 4a,b). Thus, for our whole-body analysis, we focused on the reporter lines.

## Deep learning enables whole-body analysis

**Fig. 2:**
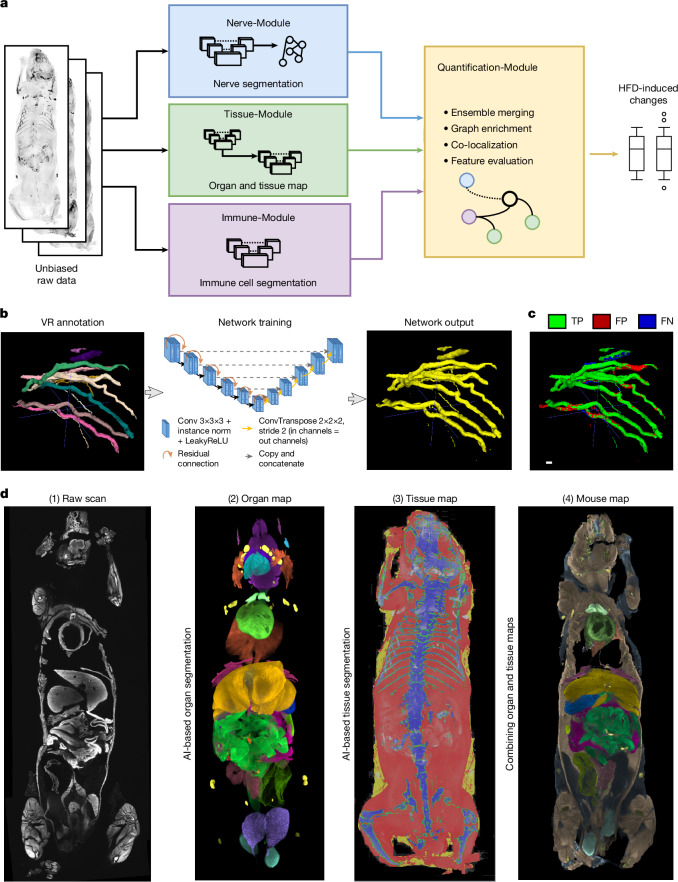
Development of an ensemble of deep-learning methods for automated segmentation of nerves, immune cells, organs and tissues. **a**, Mice that underwent vDISCO clearing, light-sheet imaging and 3D reconstruction were analysed using MouseMapper. MouseMapper comprises three modules: the Nerve-Module for deep-learning-based nerve segmentation, the Immune-Module for deep-learning-based immune cell detection and the Tissue-Module for automated organ and tissue segmentation. **b**, To train the Nerve-Module, nerves were annotated using VR. **c**, 3D qualitative evaluation of the network performance for the segmentation of nerves based on the volumetric Dice score. Areas that overlap with reference annotations (true positive, TP) are masked in green; areas with no overlap in reference annotations (false positive, FP) are masked in red. Undetected reference annotation areas (false negative, FN) are marked in blue. Scale bar, 100 μm. **d**, The Tissue-Module uses the raw image scan and segments organs (organ map) and tissues (tissue map), which can be combined to generate a whole-mouse map.

For an unbiased quantitative analysis of obesity-induced changes in whole-mouse body images, we developed MouseMapper (Fig. 2a)—an ensemble of deep-learning models that compares animals and conditions. MouseMapper comprises three main modules: (1) the Nerve-Module segments nerves in the entire mouse body, facilitating comprehensive mapping and quantitative analysis of nerve characteristics; (2) the Immune-Module segments immune cells and quantifies their distributions; and (3) the Tissue-Module maps organs and tissues to make quantitative data comparable between conditions and animals and to facilitate biological interpretation. The combination of these modules enables a comprehensive description of structural changes in nerves and immune cell distribution across the entire body (Fig. 2a).

**Extended Data Fig. 5:**
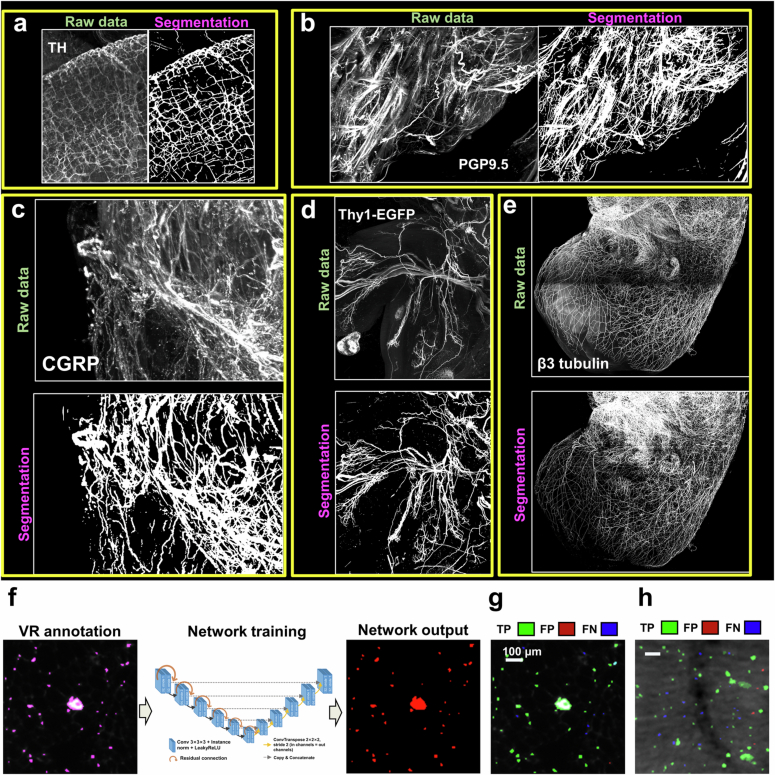
Generalizability of the MouseMapper Nerve-Module and development of the Immune Module. **a-d**, Qualitative evaluation of segmentation quality of the Nerve-Module on diverse data sets: antibody labelled mouse tissue of TH (**a**, 516 µm maximum intensity projection (MIP)), UCHL1/PGP9.5 (**b**, 3932 µm MIP), CGRP (**c**, 1396 µm MIP) and Thy1-EGFP (**d**, 11994 µm MIP)**. e**, Post-conception week 7 human embryo head antibody labelled with β tubulin (10024 µm sum of slices projection)**. f**, To train the Immune-Module, *Cd68*-eGFP+ cells were annotated using virtual reality (VR). Annotations were used to train a deep neural network, which generates the segmentation of *Cd68*-eGFP+ cells as network output. **g**,**h**, Qualitative evaluation of the network performance for the segmentation of *Cd68*-eGFP+ cells (**g**) or antibody labelled liver tissue of CD45 (**h**) based on instance Dice. Areas that overlap with reference annotations: True positives (TP); no overlap in reference annotations: false positives (FP); undetected reference annotation areas: false negatives (FN). Source data

Our models were trained in a supervised manner, with ground-truth data being generated using a 3D virtual reality (VR) annotation pipeline^7^. For the Nerve-Module, we annotated nerves in VR (84 subvolumes with 300 × 300 × 300 voxels and 8 subvolumes with approximately 1,000 × 1,000 × 1,000 voxels) from *Uchl1-*eGFP mouse scans (Fig. 2b). These curated volumes and annotations were used for training and testing nerve segmentation models in the Nerve-Module (Methods). The inherent variability in nerve morphology necessitates a segmentation model with robust generalization. We addressed this by leveraging VesselFM, a 3D foundation model pre-trained for universal blood vessel segmentation^14^. Blood vessels and nerves share fundamental topological and morphological characteristics, allowing the model’s learned generalizable features to transfer effectively to our task (Methods). After fine-tuning on our annotated nerve data, the foundation model showed superior segmentation performance compared with deep-learning models trained from scratch on our annotated data, yielding a voxel Dice score of 0.7494 (Fig. 2c, Supplementary Table 1 and Supplementary Video 8). To evaluate generalizability, we benchmarked this model on datasets acquired using different imaging and labelling strategies, including higher-resolution *Uchl1-*eGFP scans, antibody-labelled samples (UCHL1, CGRP and TH), another transgenic reporter line for neurons (*Thy1-*eGFP) and a publicly available anti-β-tubulin-stained human embryo dataset (Extended Data Fig. 5a–e). In all cases, the foundation model maintained high segmentation fidelity (voxel Dice scores of between 0.6916 and 0.7143), successfully delineating elongated axonal structures despite differences in signal intensity, labelling method and imaging scale (Supplementary Table 2 and Extended Data Fig. 5a–e).

To develop the Immune-Module, we sampled and VR-annotated *Cd68-*eGFP^+^ cells in five 256 × 256 × 256 voxel and five 128 × 128 × 128 voxel patches from *Cd68-*eGFP whole-mouse scans, representing over 500 contrast-positive cells in adipose tissue and muscle. After training and evaluation of the baselines on the test subset, we found that the fine-tuned foundation model achieved superior performance compared with other deep-learning networks (Supplementary Table 3 and Extended Data Fig. 5f–h). Moreover, our network was able to segment *Cd68-*eGFP^+^ cells in other tissues that were not part of the initial training, including the liver and gut (Supplementary Table 4), indicating the ability of the model to generalize to previously unseen tissues. We further validated its performance on independent datasets, including antibody-labelled CD45^+^ immune cells and higher-resolution *Cd68-*eGFP acquisitions. The model demonstrated strong cross-condition consistency, accurately identifying immune cells and clusters in both reporter and antibody-based datasets without retraining (Supplementary Table 5 and Extended Data Fig. 5h). Moreover, we benchmarked the Immune-Module against leading segmentation frameworks^6,10,15–17^. Our Immune-Module achieved substantially higher accuracy (voxel Dice, 0.7878; Supplementary Table 6), outperforming all existing methods (voxel Dice, 0.2140–0.5468). This demonstrates superior robustness to signal heterogeneity and dense cellular clustering, enabling reliable immune cell segmentation across diverse imaging conditions.

**Extended Data Fig. 6:**
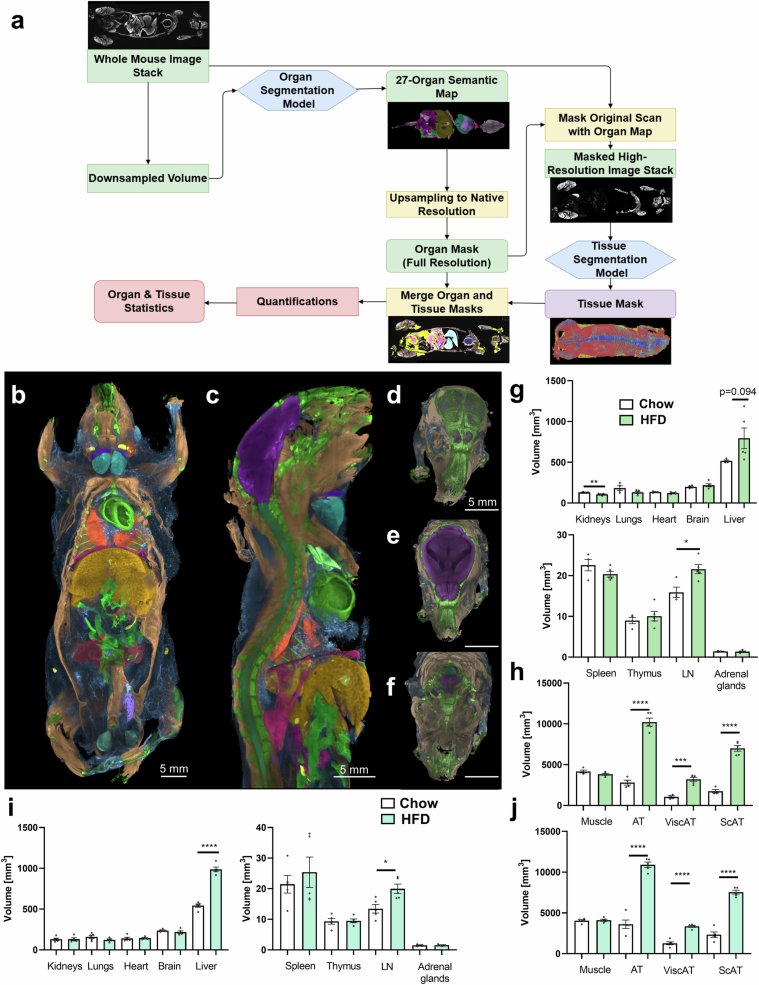
AI-based segmentation of organs and tissues with using the Tissue-Module of MouseMapper. **a**, Pipeline depicting the workflow of the organ and tissue segmentation model that was used in *Cd68*-eGFP and *Uchl1*-eGFP mouse lines. **b**,**c**, Representative HFD-fed mouse showing AI-segmented organs and tissue. Each colour represents a different organ or tissue segmented displaying b, the ventral, c, the sagittal view of the body. **d-f**, Head from a representative chow animal in rostral view with AI-segmented organs and tissues in different z-planes. **g**,**h**, Organ (g) and tissue volumes (h) segmented in *Uchl1*-eGFP mice with the pipeline shown in a **(**n = 4 chow, n = 5 HFD animals; two-tailed t-test, p-values: 0.0064 (Kidneys), 0.0118 (Lymph nodes), <0.0001 (AT, adipose tissue), 0.0002 (ViscAT), <0.0001 (ScAT)). **i**,**j**, Organ (i) and tissue (j) volumes segmented in *Cd68*-eGFP mice with the pipeline shown in a (n = 5 chow, n = 5 HFD animals; two-tailed t-test, p-values: <0.0001 (Liver), 0.0136 (Lymph nodes), <0.0001 (AT, adipose tissue), <0.0001 (ViscAT), <0.0001 (ScAT)), *p < 0.05, **p < 0.01, ***p < 0.001, ****p < 0.0001, data are represented as mean ± standard error.

Next, we engineered the deep-learning-based Tissue-Module (Fig. 2d and Extended Data Fig. 6a), which enables mapping of segmented structures (for example, nerves and immune cells) to organs and tissues, allowing for a nuanced interpretation of structural or cellular changes across conditions. To enable efficient organ mapping in the Tissue-Module, given that cell-level accuracy is of lower priority, we downsampled images of autofluorescence and the nuclear stain propidium iodide (PI) (Extended Data Fig. 6a). This enabled the processing of larger volumes by the neural network, allowing it to learn shape information while minimizing training time, inference time and memory requirements. To generate data for training and testing the models, we annotated 27 organs (Supplementary Table 7) in each of 12 whole-body mouse scans using VR. We used the annotations from eight mice to train multiple neural networks and tested their performance on the other four mice. The 3D UNet architecture, implemented in the Tissue-Module, performed best for comprehensive organ segmentation (Supplementary Table 7).

Although working with downsampled data provides highly accurate organ segmentation, adipose and muscle tissue segmentation relies on detecting differences in tissue texture, which is not well preserved in the downsampled images and requires the use of full-resolution data. Thus, we created a separate VR-annotated dataset containing representative patches from muscle, adipose tissue, bone and bone marrow in full resolution. After training, a 3D UNet showed the best performance compared with other neural networks (Supplementary Table 8). By integrating our organ and tissue models, our final Tissue-Module generates a comprehensive anatomical map of the mouse (Fig. 2d, Extended Data Fig. 6b–f, Supplementary Figs. 1–5 and Supplementary Videos 9–11). We benchmarked the Tissue-Module against state-of-the-art mouse organ segmentation frameworks^18–20^. For the three to eight organs that could be directly compared across methods, MouseMapper achieved higher Dice scores and expanded coverage to 31 organs and tissues, far beyond previous approaches (Supplementary Table 9). These results highlight MouseMapper’s superior accuracy and scalability for comprehensive whole-body organ segmentation in LSFM datasets. The volume extraction of segmented tissue and organs revealed expected increases in adipose tissue (including ViscAT and ScAT) and liver volumes in HFD-fed mice compared with in chow-fed mice (Extended Data Fig. 6g–j and Supplementary Table 10). Moreover, we found that total lymph node mass was increased after HFD feeding. Thus, this map serves as a unified reference framework, enabling precise localization of quantitative findings from cellular and anatomical analyses across different body regions.

In summary, MouseMapper represents a robust and automated AI-driven pipeline to detect and quantify system-wide perturbations in nerve structures or immune cell distributions in any size mouse body.

## Obesity-induced trigeminal nerve changes

**Fig. 3:**
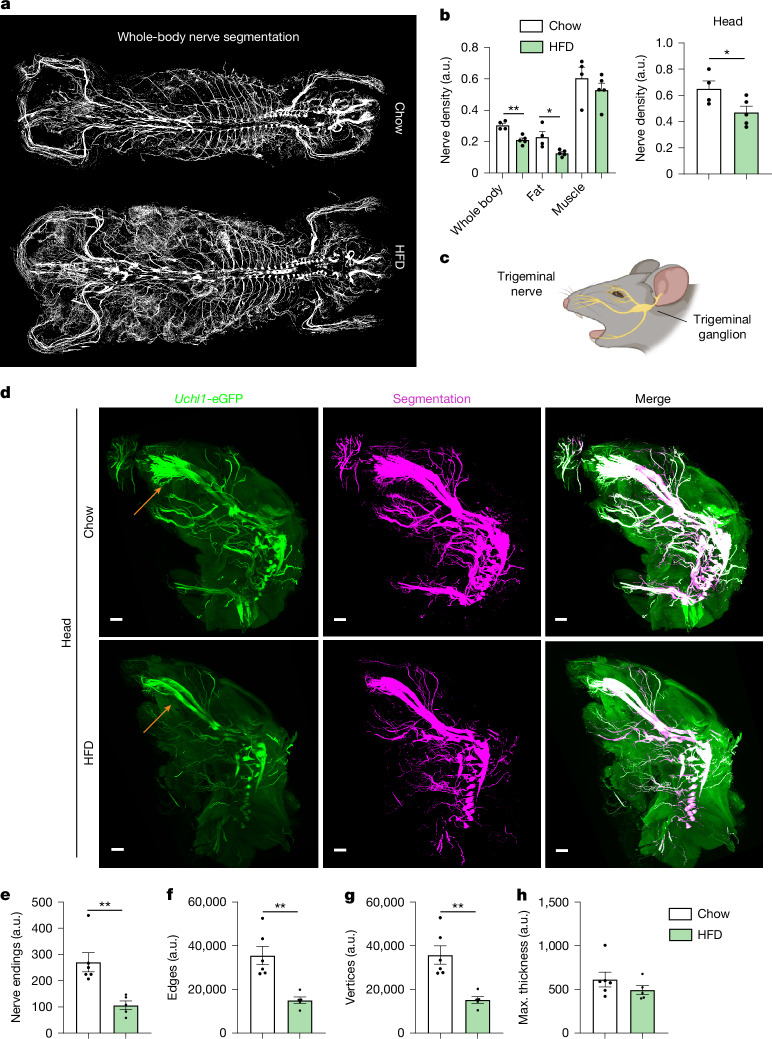
Whole-body nerve segmentation reveals structural changes in the infraorbital nerve in obesity. **a**, Representative whole-body nerve segmentation in chow and HFD-fed mice. **b**, Quantification of AI-segmented nerve densities in chow- and HFD-fed *Uchl1-*eGFP mice in whole bodies and the indicated areas. For quantification of the nerve density in the head, we masked out the brain for the analysis (*n* = 4 chow, *n* = 5 HFD mice; whole-body: two-tailed *t*-test, *P* = 0.0012; fat: two-tailed *t*-test,* P* = 0.015; head: two-tailed *t*-test, *P* = 0.0488). **c**, Schematic of a mouse head, showing the trigeminal nerve with its three branches arising from the trigeminal ganglion. The diagram was created using BioRender; Kaltenecker, D. https://biorender.com/5v5wjam. **d**, Representative heads of chow and HFD-fed mice showing *Uchl1*-eGFP^+^ nerves and AI-based segmentation of these nerves. Structural changes in the infraorbital nerve (part of the maxillary branch of the trigeminal nerve) are indicated by the orange arrow. Scale bars, 1,500 μm. **e**–**h**, Quantification of characteristics (nerve endings (**e**), edges (**f**), vertices (**g**) and thickness (**h**)) of the infraorbital nerve after graph extractions (*n* = 6 infraorbital nerves from 3 chow mice, and 5 infraorbital nerves from 3 HFD-fed mice). Statistical analysis was performed using the two-tailed Mann–Whitney *U*-test, *P* = 0.0043 (**e**); two-tailed *t*-test, *P* = 0.0022 (**f**); and two-tailed* t*-test, *P* = 0.0022 (**g**). *P < 0.05, **P < 0.01. Data are mean ± s.e.m. Source data

**Extended Data Fig. 7:**
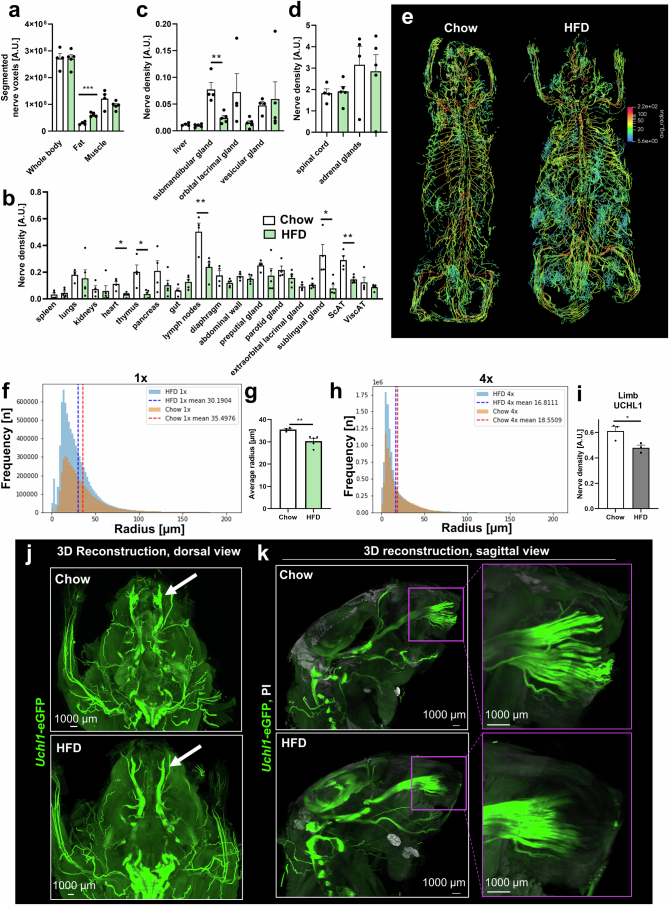
Quantification of segmented nerves and 3D visualization of *Uchl1*-eGFP mouse heads after vDISCO clearing, imaging, and reconstruction. **a**, Total nerve voxels of segmented nerves in the whole body, fat and muscle (n = 4 chow, n = 5 HFD animals; two-tailed t-test, p-value: 0.0002 (Fat). **b-d**, Quantification of nerve density in indicated organs/tissues. **(**n = 4 chow, n = 5 HFD animals; two-tailed t-test, p-value: 0.0051 (heart), 0.0111 (thymus), 0.0067 (lymph nodes), 0.0147 (sublingual gland), 0.0034 (ScAT), 0.004 (submandibular gland); *p < 0.05, **p < 0.01, ***p < 0.001. **e**, Representative whole-body nerve graphs extracted from segmentations colour-coded by local radius. For visualization, all radii below 5.6 µm were grouped into the 5.6 µm category. **f**, Histogram of nerve radii extracted of 1× graphs from all mice (n = 4 chow, n = 5 HFD animals). **g**, Average nerve-segment radius from 1× datasets (n = 4 chow, n = 5 HFD animals; two-tailed t-test, p-value = 0.0054). **h**, Histogram of nerve radii extracted from 4× datasets from one chow and one HFD mouse scan. **i**, Limb nerve density based on PGP9.5 antibody staining (n = 3/group; two-tailed t-test, p-value = 0.0386). **j**, Dorsal view of 3D reconstructions of heads of chow- and HFD-fed mice showing *Uchl1*-eGFP+ nerves. White arrows indicate structural changes in the infraorbital nerve. **k**, Sagittal view of 3D reconstructions of heads of chow- and HFD-fed mice showing *Uchl1*-eGFP+ nerves with zoomed-in views of the intricate nerve structures. The left side of the head was clipped away for optimizing visualization. Data are represented as mean ± standard error. Source data

Obesity is associated with various neuronal malfunctions, including peripheral neuropathies, yet a comprehensive characterization of obesity-induced changes in peripheral nerves on the whole-body scale is lacking. Toward this goal, we applied MouseMapper with the Nerve-Module and Tissue-Module to whole-body scans of normal and obese *Uchl1-*eGFP mice (Fig. 1b), generating whole-body segmentation maps of the peripheral nervous system (Fig. 3a). While total nerve voxels were similar between the groups (Extended Data Fig. 7a), whole-body nerve density, which accounts for differences in body sizes between lean and obese mice, was decreased after HFD feeding (Fig. 3b). Moreover, we mapped the nerve segmentation to the tissues covered by the Tissue-Module (Extended Data Fig. 7a–d). In adipose tissue, where HFD feeding induces marked tissue expansion, total nerve voxels were increased in obesity, consistent with the larger fat mass (Extended Data Fig. 7a). Yet, analogous to the whole-body analysis, nerve density in adipose tissue was decreased (Fig. 3b), demonstrating that innervation does not scale proportionally with tissue growth and is relatively reduced in obesity.

We also converted the nerve segmentation into whole-body nerve graphs, enabling quantification of local radii along individual nerve fibre bundles (Extended Data Fig. 7e). Radius measurements revealed a left-shift in nerve calibre distribution and a reduction in average nerve bundle radius in obese mice compared with in the lean controls (Extended Data Fig. 7f,g). This shift towards smaller diameters was also observed in the higher-resolution datasets (Extended Data Fig. 7h), confirming that the reduction in bundle size persists even when finer terminal branches are resolved. Furthermore, we detected significant reductions in nerve density in limbs of obese mice that were antibody labelled with UCHL1 (Extended Data Figs. 3a,b and  7i).

Notably, we observed a significant decrease in nerves located in the head (Fig. 3b). Most prominently, we observed structural alterations in the infraorbital nerves that innervate the whisker pad after HFD-induced obesity (Fig. 3c,d and Extended Data Fig. 7j,k). The infraorbital nerve, a key branch of the maxillary division of the trigeminal nerve, is essential for facial sensory perception, facilitating whisker-mediated tactile exploration and environmental sensing. To quantify the spatial structure of these nerves in more detail, we extracted graphs from the binary nerve segmentation, measuring nerve thickness and the number of nerve endings, but also quantifying the complexity of the nerve network by determining the number and length of edges and the number of nodes/vertices^21^. Here, nodes/vertices represent the points of intersection or branching within the nerve network and edges represent the connections between these points along the nerve pathways. Quantification of nerve segmentation graphs showed that the number of nerve endings, edges and vertices were reduced by 60.7%, 57.8% and 57.6%, respectively, in HFD-fed obese mice (Fig. 3e–g). Notably, the thickness of the infraorbital nerve was similar between the chow- and HFD-fed mice (Fig. 3h), indicating defects in axonal extensions away from the ganglia rather than a general degeneration of the nerve.

**Fig. 4:**
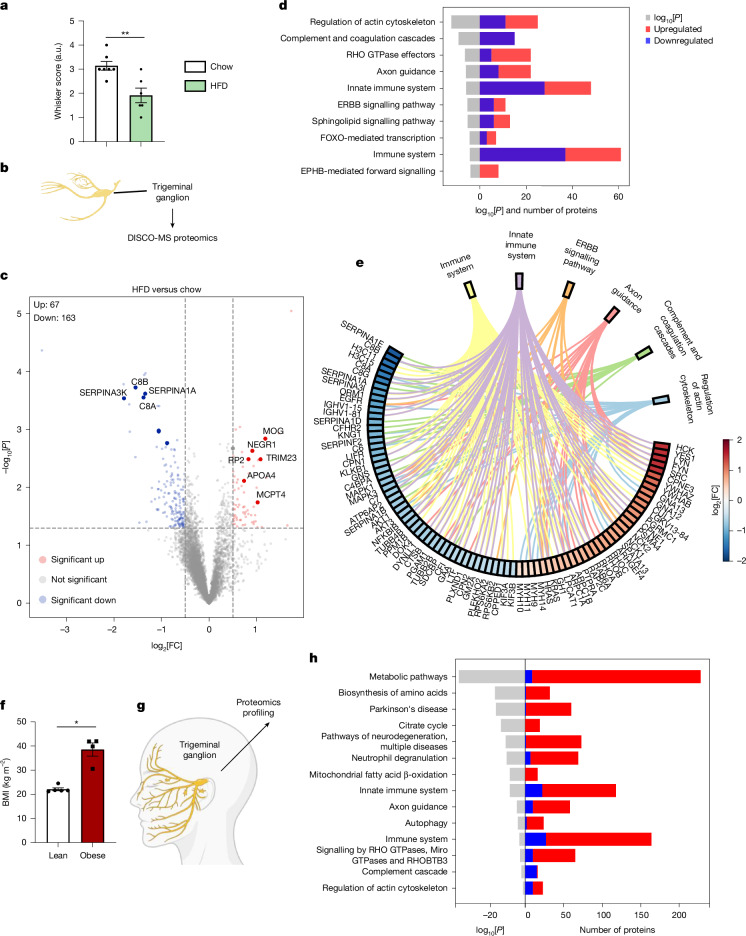
Structural changes in the infraorbital nerve are associated with changes in the trigeminal ganglion proteome. **a**, Functional assessment of the response after whisker stimulation. *n* = 7 (chow), *n* = 6 (HFD). Statistical analysis was performed using two-tailed *t*-tests; *P* = 0.0040. Data are mean ± s.e.m. **b**, Samples for spatial proteomics profiling were extracted from the trigeminal ganglia of *Uchl1-*eGFP mice. The diagram was created using BioRender; Kaltenecker, D. https://biorender.com/5v5wjam. **c**, Differentially regulated proteins in chow versus HFD-fed mice. *n* = 6 chow trigeminal ganglia (right and left, from 3 mice), *n* = 6 HFD trigeminal ganglia (right and left, from 3 mice). **d**, Pathway analysis showing differentially regulated pathways. The grey bar on the left of the plot represents the log_10_-transformed *P* value of each pathway, whereas the right side of the plot shows the number of proteins significantly different in each pathway, where red represents the number of upregulated proteins and blue represents the number of downregulated proteins. **e**, A subset of differentially regulated pathways and the corresponding proteins. **f**,**g**, The BMI of lean individuals (BMI < 25) and individuals with obesity (BMI > 30) (**f**; *n* = 5 (lean), *n* = 4 (obesity); two-tailed Mann–Whitney *U*-test, *P* = 0.0159) from which trigeminal ganglia were isolated post-mortem for proteomic profiling (**g**). **h**, Pathway analysis showing differentially regulated pathways in human trigeminal ganglia. *n* = 15 (lean; 3 regions of interest sampled per individual from 5 individuals total) and *n* = 12 (obesity; 3 regions of interest per individual from 4 individuals total). The colour scheme is as described for **d**. Source data

To assess the functional implications of these structural changes, we performed whisker-stimulation tests and found that obese mice exhibited a diminished response to whisker stimulation (Fig. 4a). This finding suggests that obesity-induced structural changes in facial branches of the trigeminal nerve may contribute to sensory dysfunction, highlighting the potential importance of our observations.

## Trigeminal ganglion proteomic alterations

**Extended Data Fig. 8:**
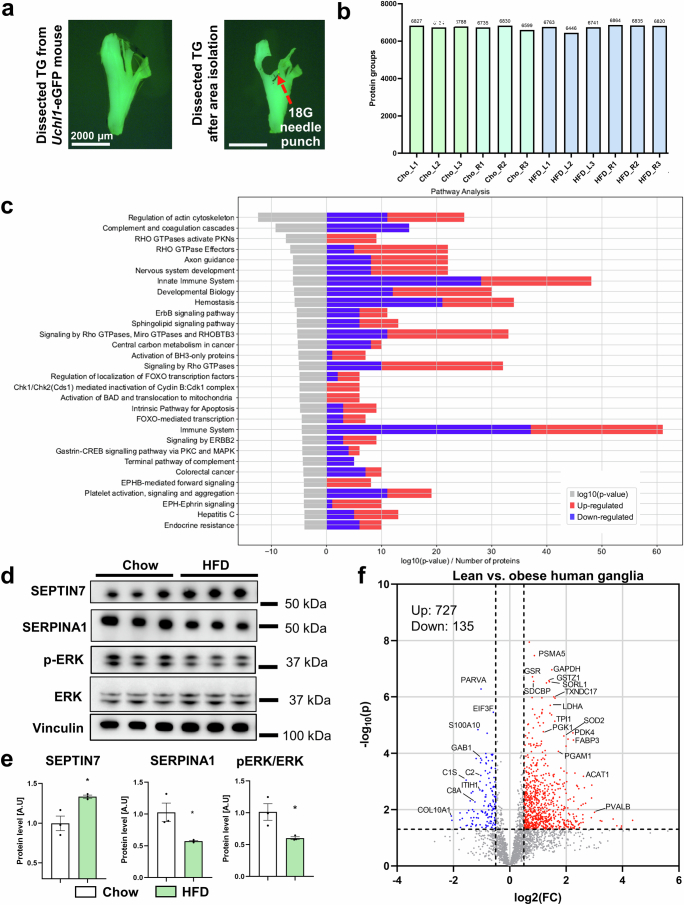
Trigeminal ganglion proteome differences in chow vs high-fat diet fed animals. **a**, Dissected trigeminal nerve from *Uchl1*-eGFP mouse before (left) and after (right) puncture with the 18 G needle. Red circle indicates the excised region that was subjected to proteomic analysis. **b**, Number of protein groups detected in each proteomic sample. Cho stands for chow group and HFD stands for high-fat diet-fed group. L stands for the left trigeminal ganglion and R stands for the right trigeminal ganglion. **c**, Pathway analysis from the TG of chow vs HFD is depicted. The pathways that are affected are shown on the y-axis of the plot. Grey bar on the left side of the plot represents the log10 of p-value of each pathway whereas the right side of the plot depicts the number of proteins significantly different in each pathway, red representing the number of up-regulated proteins and blue representing the number of down-regulated proteins. **d**, Western blot of protein lysates from trigeminal ganglia of chow and HFD-fed mice to validate proteins that were differentially expressed in the proteomic analysis: SEPTIN7, SERPINA1, p-ERK, and ERK proteins using western blot. Vinculin is shown as representative loading control (n = 3/group). **e**, Quantification of SEPTIN7, SERPINA1 and pERK/ERK protein levels detected in chow vs HFD groups (n = 3/group, two-tailed t-test, p-values: 0.0229 (SEPTIN7), 0.0355 (SERPINA1), 0.0357 (p-ERK/ERK). For source data, see Supplementary Fig. 6. **f**, Volcano plot showing differentially regulated proteins in the trigeminal ganglion of lean and obese humans (n = 15 lean (3 regions of interest sampled per individual from 5 individuals total); n = 12 obese (3 regions of interest per individual from 4 individuals total)). Data are represented as mean ± standard error. Source data

Next, to investigate the molecular mechanisms underlying these changes in the infraorbital nerve, we performed spatial proteomics profiling^11^ of the trigeminal ganglia, the origin of the infraorbital nerve and the location of their neuronal cell bodies. We dissected the trigeminal ganglia of chow and HFD-fed mice that we imaged above, collected 18G-needle punch-sized samples, and analysed them using the mass spectrometry (MS)-based proteomics (Fig. 4b and Extended Data Fig. 8a). We identified more than 6,000 total proteins in each sample (Extended Data Fig. 8b). Among them, 230 were differentially regulated (67 upregulated, 163 downregulated; Fig. 4c) in the trigeminal ganglia between chow and HFD-fed mice.

Pathway analysis revealed multiple differentially regulated pathways in HFD-fed mice, including regulation of actin cytoskeleton, RHO GTPase effectors and axon guidance (Fig. 4d,e and Extended Data Fig. 8c). This could indicate disruptions in actin dynamics, which is essential for maintaining axonal structure and function. Moreover, significantly regulated pathways included complement and coagulation cascade, ERB signalling and sphingolipid signalling pathways that are involved in inflammation and cellular stress response, among other processes, which also fits to the dysregulation of many general and innate immune pathways (Fig. 4d,e and Extended Data Fig. 8c).

Among downregulated proteins, we identified multiple members of the SERPIN-A family (Fig. 4e). SERPINA1 has anti-inflammatory properties, especially linked to neutrophils (by inhibiting neutrophil elastase) and is known to protect from tissue damage^22^. SERPINA3 is an inhibitor of cathepsin G, another protease important in neutrophil related immune responses and implicated in inflammation related tissue damage^23^. Downregulation of SERPINA proteins might lead to a reduced ability to control inflammation-induced tissue damage and degradation of structural proteins essential for nerve integrity. We validated the proteomic findings using western blotting for decreased SERPINA1 expression, ERK activation (ERBB signalling) and increased expression of the GTPase SEPTIN7 (actin cytoskeleton regulation) (Extended Data Fig. 8d,e).

To assess whether the molecular alterations identified in mice reflect conserved changes in humans, we further analysed post-mortem trigeminal ganglia from lean individuals (body mass index (BMI) < 25) and individuals with obesity (BMI > 30) (Fig. 4f,g and Supplementary Table 11). Proteomic profiling revealed that obesity was associated with extensive remodelling of the trigeminal proteome, including differential regulation of pathways linked to axon guidance, neurodegeneration and the regulation of the actin cytoskeleton, thereby mirroring our findings in obese mice (Fig. 4h, Extended Data Fig. 8f and Supplementary Table 12). Moreover, expectedly, numerous pathways related to metabolism were differentially regulated in humans with obesity, including the biosynthesis of amino acids, the citrate cycle or pyruvate metabolism (Supplementary Table 12).

Together, spatial molecular profiling of the trigeminal ganglion in HFD-fed mice, revealed by our MouseMapper deep-learning ensemble, showed significant proteomic alterations that were recapitulated in human post-mortem tissue. Among these, we identified dysregulated pathways related to axon growth and remodelling, and inflammation, which could explain structural changes in the infraorbital nerves.

## Whole-body-wide inflammation in obesity

**Fig. 5:**
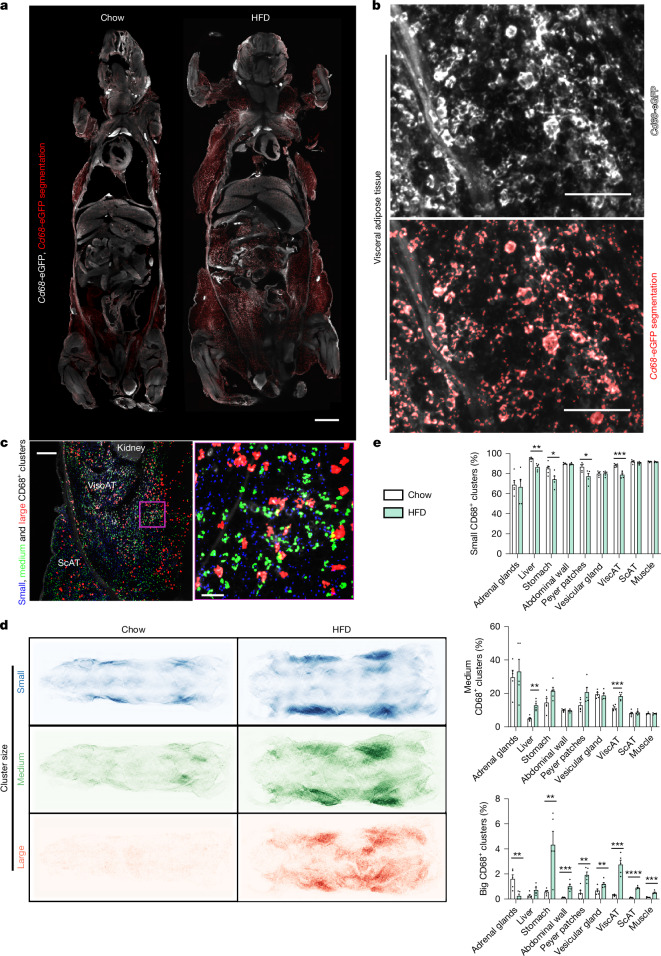
Whole-body-wide inflammation in obesity. **a**, Representative images of *Cd68-*eGFP mice; AI-based segmentation of the *Cd68-*eGFP signal is overlaid in red. *n* = 5 (chow), *n* = 5 (HFD). **b**, Representative* z*-projection (300 µm) view showing ViscAT with *Cd68-*eGFP (top) and AI-based segmentation (bottom) of an obese mouse. *n* = 5 (chow), *n* = 5 (HFD). **c**, Representative *z*-projection (300 µm) showing segmented cells that were grouped into three size clusters: small (blue), medium (green) and large (red). Clusters located at different depths along the projection axis can appear to overlap in the image, even though they are spatially distinct in 3D space. *n* = 5 (chow), *n* = 5 (HFD). **d**, Whole-body *Cd68-*eGFP segmentation results in representative chow and HFD-fed mice showing small, medium and large cluster densities. *n* = 5 (chow), *n* = 5 (HFD). **e**, Quantification of small, medium and large cluster proportions in the indicated organs and tissues. *n* = 5 (chow), *n* = 5 (HFD). Statistical analysis was performed using two-tailed *t*-tests for all comparisons except for adrenal glands and muscle big CD68^+^ clusters, for which a two-tailed Mann–Whitney *U*-test was used. Exact *P* values were as follows: small CD68^+^ clusters: *P* = 0.0015 (liver), 0.0341 (stomach), *P* = 0.026 (peyer patches), *P* = 0.0003 (ViscAT); medium CD68^+^ clusters: *P* = 0.001 (liver), *P* = 0.0006 (ViscAT); big CD68^+^ clusters: *P* = 0.0079 (adrenal glands), *P* = 0.0091 (stomach), *P* = 0.0007 (abdominal wall), *P* = 0.0029 (peyer patches), *P* = 0.0303 (vesicular gland), *P* = 0.0001 (ViscAT), *P *< 0.0001 (ScAT), 0.0079 (muscle). ***P < 0.001, ****P < 0.0001. Data are mean ± s.e.m. Scale bars, 5,000 μm (**a**), 1,000 μm (**c**, left), 500 μm (**b**) and 200 μm (**c**, right). Source data

Chronic inflammation is a major hallmark of obesity, intricately linked to the development of various chronic diseases throughout the body. The systemic nature of obesity-induced inflammation underscores the critical importance of understanding which tissues and organs are affected in obese animals and to what degree. To study the spatial context of inflammation in obesity, we applied MouseMapper using the Immune-Module and Tissue-Module to whole-body scans of lean and obese *Cd68-*eGFP mice (Fig. 5a,b).

**Extended Data Fig. 9:**
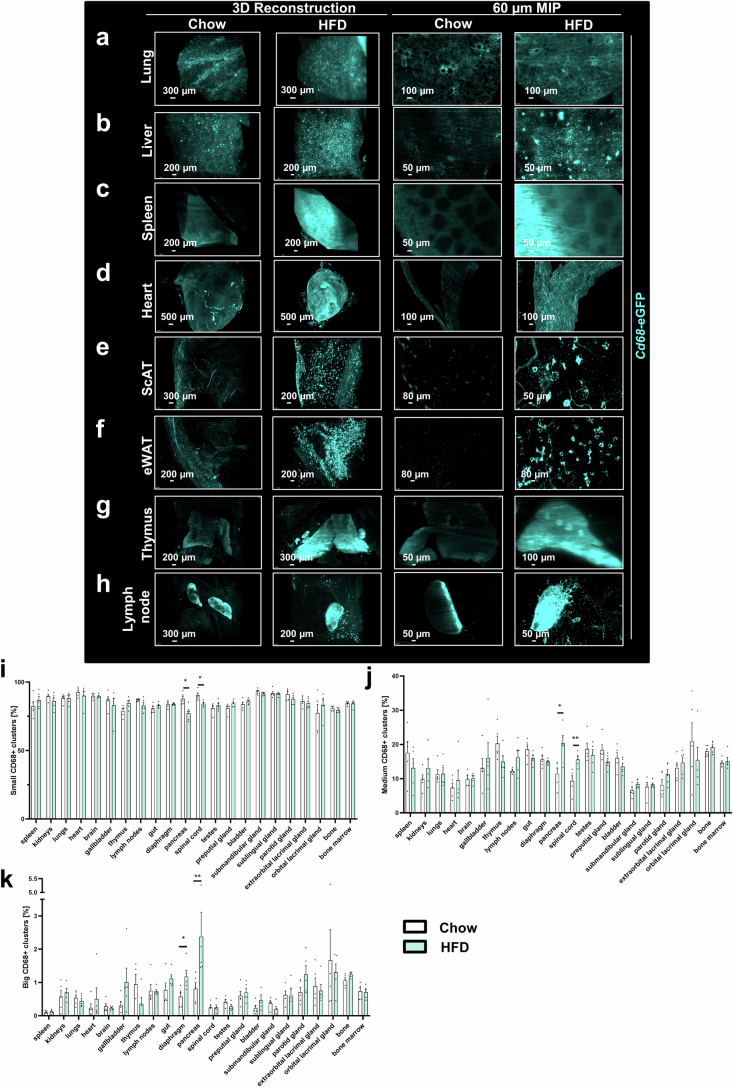
High resolution *Cd68*-EGFP organ comparison and quantification across mapped tissue. **a-h**, Representative 3D reconstruction and 2D 60 µm thick maximum intensity projections (MIP) of (a) lung, (b) liver, (c) spleen, (d) heart, (e) scWAT, (f) eWAT, (g) thymus and (h) accessory axillary lymph node from the *Cd68*-eGFP mouse line after vDISCO and light sheet imaging for chow and high fat diet fed animals. The left two columns represent the 3D reconstruction of the organs and the right two columns represent the MIPs. For each representation chow fed animals are shown on the left column and the high-fat diet fed animals are shown in the right column. The images are acquired with a 4x objective and are visualized using the same brightness and contrast settings. **i-k**, Quantification of small, medium and big cluster proportions in indicated organs and tissues (n = 5/group, small CD68+ clusters: two-tailed t-test, p-values: 0.0101 (pancreas), 0.0108 (spinal cord); medium CD68+ clusters: two-tailed t-test, p-values: 0.0135 (pancreas), 0.0088 (spinal cord); large CD68+ clusters: two-tailed Mann-Whitney test, p = 0.0079 (pancreas), two-tailed t-test, p = 0.0322 (diaphragm)), *p < 0.05, **p < 0.01, data are represented as mean ± standard error. Source data

The *Cd68-*eGFP^+^ immune cells were visible as round, cluster-like structures in tissues, including adipose tissue, liver, skeletal muscle and the peritoneum (abdominal wall; Extended Data Fig. 9a–h and Supplementary Video 6). The size of immune cell clusters can indicate the inflammatory state within tissues, with larger clusters correlating with a more activated and pro-inflammatory state^24^. Thus, we generated inflammation maps of *Cd68-*eGFP^+^ immune cells and grouped them into three different sizes of clusters: small (up to 6 cells), medium (6–60 cells) and large (60+ cells) (Fig. 5c,d) based on the high-resolution cell-level scans with a higher-NA ×4 objective. Using these categories, we analysed the density of the three different classes of *Cd68-*eGFP^+^ clusters (Fig. 5e and Supplementary Video 12) and found marked decreases in small-cluster portions within the liver, ViscAT and stomach after HFD feeding, whereas this category remained unchanged in the ScAT, peritoneum and muscle (Fig. 5e and Extended Data Fig. 9i). Conversely, the proportion of medium-sized clusters showed an increase specifically in the liver and ViscAT, highlighting a shift from small to medium clusters in these tissues (Fig. 5e and Extended Data Fig. 9j). Moreover, we observed significant increases in large clusters in several areas, including the ScAT, ViscAT, muscle, stomach and abdominal wall, signifying an intensification of inflammatory activity and immune cell involvement in obesity (Fig. 5e and Extended Data Fig. 9k).

**Extended Data Fig. 10:**
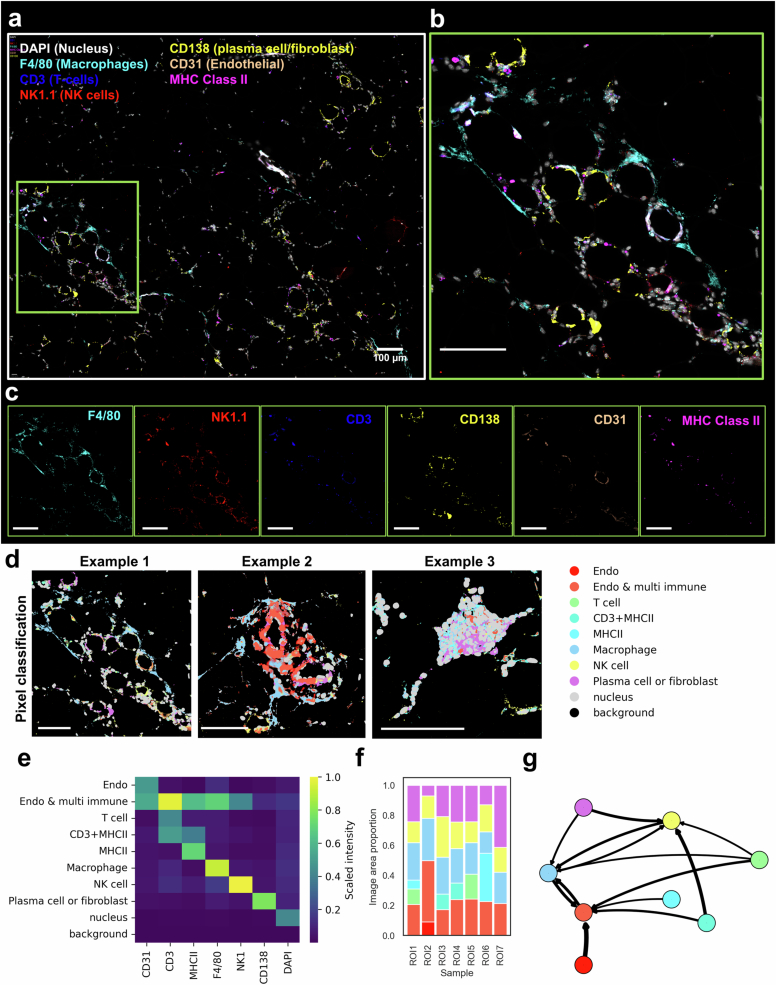
Multiplex imaging and pixel-level analysis of immune and endothelial architecture. **a**, Representative multiplex immunofluorescence staining showing one region of interest with indicated markers in a section of obese epididymal white adipose tissue (n = 7 region of interests (ROI)). **b**,**c**, Zoom-in view of the boxed region in (a) highlighting multicellular interfaces and local tissue architecture (b) and individual marker channels (**c**). **d**, Self-organizing map (SOM) and Leiden clustering-based pixel classification illustrating segmentation according to marker combinations in an example from same field as in b), example 2 (containing a prominent vessel) and example 3 (big cell accumulation) from different ROIs. **e**, Mean fluorescence intensities in each pixel class across all channels used for pixel classification scaled by the maximum mean intensity, demonstrating marker-specific segregation. **f**, Quantitative pixel composition analysis from seven independent regions of interest (ROI1–ROI7) showing proportional contributions of endothelial and immune cell classes to total image area. **g**, Spatial proximity network derived from pixel-level co-occurrence analysis showing frequent interactions between analysed cell types. The thickness of each arrow indicates how frequently pixels of the target class (arrow tip) occur next to pixels of the source class (arrow origin).

We performed multiplex labelling for five immune cell markers and endothelial cells on ViscAT sections to assess which other cell types cluster with macrophages in obese mice (Extended Data Fig. 10a–c). Images were segmented at the pixel level for all markers, resulting in the classification of pixels into 11 distinct subclasses (including nuclei and background) (Extended Data Fig. 10d,e), defined by characteristic fluorescence intensity profiles across the multiplex panel. Partial co-localization of CD3 and MHC-II signals indicated close spatial interactions between T cells and antigen-presenting macrophages, dendritic cells or B cells within macrophage-rich clusters. Quantitative pixel composition (Extended Data Fig. 10f) revealed that macrophages are consistently represented components of local immune aggregates, with variable regional enrichment of T cells, natural killer (NK) cells, CD138^+^ cells and MHC-II^+^ antigen-presenting cells. Spatial proximity analysis (Extended Data Fig. 10g) revealed that macrophages were most frequently co-localized with T cells (CD3^+^), NK cells (NK1.1^+^) and endothelial cells (CD31^+^), indicating the formation of perivascular immune hubs. By contrast, CD138^+^ cells (plasma cells or fibroblasts) remained largely separated from these multicellular aggregates, indicating distinct spatial compartmentalization (Extended Data Fig. 10g).

Together, direct visualization of *Cd68-*eGFP^+^ cells revealed widespread increases throughout the body after HFD-induced obesity. Using the Tissue-Module combined with the AI-based macrophage detection of the Immune-Module, we quantified shifts in CD68^+^ cluster sizes, confirming elevated inflammatory states across tissues in response to a HFD-induced obesity and providing detailed spatial information.

## Discussion

In this study, we developed MouseMapper, a deep-learning framework for the comprehensive, end-to-end analysis of perturbations in whole-body systems. Our approach enables 3D organ and tissue mapping of structural alterations to study disease-induced changes in biological systems down to cellular resolutions at the whole-body scale without pre-defining specific tissue regions. MouseMapper can faithfully segment elongated nerve structures over centimetres in whole-mouse bodies. It can also identify and analyse immune cells from single cells to clusters of hundreds of cells in 3D.

A key strength of MouseMapper lies in its powerful deep-learning ensemble, trained on datasets coming from cell-level imaging of whole-mouse bodies. This includes nerves traced over long distances using VR in entire mammalian bodies at high resolution, a dataset that presents unique challenges due to the diverse tissue backgrounds encountered across the body, from muscle to bones to various organs. In contrast to previous methods for whole-body analysis^6,10,18^, MouseMapper performs true 3D sliding-window inference on uncropped, multiterabyte datasets, rather than relying on 2D projections or precropped volumes, ensuring unbiased system-wide screening of anatomical changes for elongated structures. Importantly, the nerve and immune modules use a foundation model, in which networks were pretrained on many large-scale volumetric biomedical datasets and fine-tuned for whole-body LSFM images. This design enhances segmentation fidelity, robustness and cross-dataset generalizability across different resolutions and antibody labelling strategies. Previous methods such as DeepMACT^6^ (tumour metastasis quantification in manually annotated organs), DELiVR^7^ (neuronal activation and microglial somata quantification and mapping in whole brain), AIMOS^18^ (segmentation of six organs for CT/LSFM) and SCP-Nano^10^ (nanocarrier targeted cell quantification in manually annotated organs) targeted a limited set of biological systems. MouseMapper provides an integrated, multisystem analysis platform with the ability to segment peripheral nerves, immune cells and tissue compartments across the entire adult mouse body. Its modular architecture, comprising dedicated nerve, immune and tissue segmentation modules, was specifically designed for complex, systemic disease models such as obesity, in which structural and cellular changes span multiple organs and systems.

At the methodological level, MouseMapper includes the first deep-learning pipeline for segmentation of elongated nerve structures across full-body datasets. It is also the first AI-based framework for muscle and adipose tissue segmentation at whole-body resolution, and achieves by far the largest number of automatically segmented organs and tissue (*n* = 31) reported for LSFM datasets, far exceeding previous methods such as AIMOS (six organs). Notably, our Tissue-Module provides crucial anatomical context that can localize identified changes within specific organs and tissues and can serve as a common reference framework for other whole-body data. A limitation of contemporary deep-learning methods is that full generalizability across disparate imaging modalities is not yet attainable, and MouseMapper may require additional fine-tuning for optimal performance on new datasets. To promote further research and development in this field, we are making our data and algorithms publicly available.

Despite the major technical advances introduced here, several limitations remain inherent to whole-body light-sheet microscopy. First, the achievable imaging resolution represents a key constraint for resolving the smallest parenchymal nerve fibres and subcellular structures. Our standard configuration (1.1× objective, NA 0.1; lateral resolution of around 5.9 µm) provides an optimal balance between comprehensive whole-body coverage and cellular-level detail, but cannot fully resolve the thin axons (sub-micrometre to a few micrometres). These fine elements appear as bundled or merged signals at this voxel size and, therefore, detailed reconstruction of terminal arbours or synaptic boutons remains beyond the scope of whole-body imaging. To partially overcome this limitation, we implemented a higher-resolution 4× (NA 0.35; lateral resolution of 1.62 µm) and high-speed imaging approach on the light-sheet microscope, which reduced the whole-body acquisition time from nearly 2 weeks to approximately 20 h. This enabled more accurate detection of thinner axons and isolated immune cells across large specimens. However, this enhancement comes at the cost of substantially increased data volumes (up to 50 TB per mouse), highlighting the trade-off between spatial resolution and scalability. Future advances in optics, adaptive sampling strategies, and data handling and processing technologies may help bridge this gap, enabling sub-micrometre resolution across macroscopic volumes.

*Uchl1-*eGFP mice provided a uniform and high-contrast signal necessary for unbiased whole-body nerve reconstruction and AI-based quantification. Across whole-body datasets, MouseMapper revealed a marked reduction in nerve density in obese *Uchl1-*eGFP mice. Among other tissues, this decrease was particularly evident in adipose tissue, consistent with previous reports documenting decreased adipose-tissue innervation in obesity^25–27^. Nevertheless, the *Uchl1-*eGFP mouse line expresses GFP from the *Uchl1* promoter and does not tag the endogenous protein. This can lead to differences in expression patterns, which can, for example, cause absence of GFP expression in some nerves that express the endogenous UCHL1 protein or weak labelling of terminal fibres. To complement this, we tested UCHL1 immunolabelling at the whole-body scale^28^. Although antibody penetration was less homogeneous in obese mice, probably due to increased tissue thickness and lipid content, the staining remained robust in specific regions. In the limbs, UCHL1 data likewise supported a reduction in nerve density in obese mice. These findings highlight that MouseMapper can detect biologically meaningful patterns of obesity-associated neuropathy despite incomplete visualization of the smallest terminal fibres.

Our key biological finding includes structural changes in the infraorbital nerve of obese mice. The infraorbital nerve belongs to the facial trigeminal nerve, which consists of three branches that convey sensory signals from the face to the spinal cord through the trigeminal ganglion^29^. Our data reveal a previously unrecognized impact of obesity on the trigeminal facial nerve structure that was linked to functional sensory deficits in whisker stimulation. The reductions in nerve endings and network complexity suggest a potential mechanism for sensory alterations in obesity, including the reduced sensitivity to whisker stimulation observed by us and the aberrant sensory and pain processing previously observed in obese mice^30–32^. The proteomic changes identified in the trigeminal ganglion offer insights into the molecular underpinnings of these neuronal changes. In this regard, the observed changes in pathways related to cytoskeletal regulation and axon guidance in the trigeminal ganglion could potentially explain the observed changes in infraorbital nerve structure, as both are essential for structural plasticity^33,34^. Notably, we found that key molecular signatures of these pathways were conserved in trigeminal ganglia from obese humans, providing a translational bridge for our findings. The proteomic changes related to inflammation underscore the link between obesity and neuroinflammation^35^. These insights could pave the way for therapeutic approaches targeting neuroinflammation and cytoskeletal integrity in obesity and related conditions. Notably, these changes probably reflect a combination of neuronal and non-neuronal responses, as neurons comprise only a fraction of cells in the ganglia^36^.

Our data using *Cd68-*eGFP mice support previous findings that obesity is associated with chronic inflammation^37^, as we observed increased expression numbers of *Cd68-*eGFP^+^ cells throughout the mouse. Consistent with previous reports, our data confirm a more pronounced accumulation of large *Cd68-*eGFP^+^ clusters in the visceral fat compared with in the subcutaneous fat^38^. Our whole-body mapping approach adds a comprehensive spatial view of obesity-induced inflammation, revealing tissue-specific patterns of macrophage accumulations, which can reflect both inflammatory and remodelling states^39^ in our HFD-induced obesity model. To add cellular and molecular depth to these maps, we used higher-resolution ×4 imaging to better estimate cell numbers within clusters and applied multiplexed immunofluorescence to characterize the diverse immune cell populations, including T cells, that co-accumulate within these sites.

We made the whole-mouse-body maps available online (https://discotechnologies.org/MouseMapper/), where scientists can easily scroll through large datasets of HFD-fed versus chow-fed mice to investigate neuronal and immune cell alterations. Researchers can quickly identify obesity-induced changes in their tissues/organs of interest and explore potential connections with other body systems. These online maps can save time and resources and provide a broader context for understanding localized changes within the global landscape of obesity-induced alterations.

In conclusion, MouseMapper provides a powerful and scalable blueprint for the holistic analysis of complex biological phenomena in 3D. We revealed site-specific neuropathies in obesity, linked them to functional and molecular changes and demonstrated their relevance to human pathology. The pipeline can be easily adapted to other complex diseases and body-wide systems, such as the lymphatic and vascular system. In combination with spatial proteomics analysis of hotspots of structural alterations, MouseMapper facilitates the identification of potential therapeutic targets to reverse or prevent pathological changes. MouseMapper therefore provides a blueprint for the holistic analysis of complex biological phenomena in 3D.

## Methods

### Animals

Male *Uchl1-*eGFP and *Cd68-*eGFP mice (aged 8 weeks) on the C57BL/6J background or wild-type C57BL/6J mice were fed either a chow diet or a high-fat diet (60% fat, D12492i from Research Diets) for 16–18 weeks ad libitum. Mice were maintained on a 12 h–12 h light–dark cycle. The set points in the animal room were adjusted to 20–24 °C temperature and 45–65% humidity. Body composition was determined using an EchoMRI-100H system (EchoMRI). For insulin-tolerance tests, mice were fasted for 6 h and intraperitoneally (i.p.) injected with 0.75 U kg^−1^ insulin. Blood glucose was measured from the tail vein at the indicated timepoints using glucose test stripes. Mice were euthanized after deep anaesthesia with a mix of ketamine and xylazine, followed by intracardiac perfusion with heparinized PBS (10 U ml^−1^ heparin) and by a perfusion with 4% paraformaldehyde (PFA). Mice were post-fixed overnight in 4% PFA and subsequently washed five times with PBS shaking (300 rpm) at room temperature for 1 h for each wash step. Animal experimentation was performed in accordance with the European Union directives and the German animal welfare act (Tierschutzgesetz). They have been approved by the state ethics committee and the government of Upper Bavaria (ROB-55.2-2532.Vet_02-21-133, ROB-55.2-2532.Vet_02-16-117, ROB-55.2-2532.Vet_02-17-49, ROB-55.2-2532.Vet_02-19-166).

### Human participants

Trigeminal ganglion samples were dissected post-mortem from body donors at the Institute of Anatomy, University of Leipzig, Germany and fixed in 4% Histofix. Body donors gave their informed and written consent to explore the cadavers for research and educational purposes (ethical approval number 129/21-ck, Medizinische Fakultät Ethik-Kommission). The participants were divided into lean (BMI < 25) or obese (BMI > 30). Data on age and sex can be found in Supplementary Table 11. We dissected three regions of interest from each trigeminal ganglion per individual for proteomic profiling.

### Whisker stimulation test

The whisker test paradigm was adapted from the methods described previously^40–43^ and the Neuroscore test^44^. To avoid introducing confounding variables, mice were kept in their original cages. A cotton swab with a wooden end was used to administer the test. Initially, the cotton swab was presented in front of the mouse’s head and allowed to touch it. This was followed by four consecutive strokes, first to the whiskers on the right side and then on the left side of the face. The response to the cotton swab stimulation was evaluated using a modified whisker score test. A normal behavioural response to the stimulation, such as turning the head towards or away from the cotton swab or initiating grooming, was assigned a score of one. A lack of response to the stimulation was assigned a score of zero. Both sides of the face were stimulated four times, and the scores were recorded by a blinded evaluator. The maximum whisker score was 8, in which mice would have responded to all stimuli. The total score was then averaged for both sides. High scores (3–4) indicated normal responses to the stimulation, while low scores (0–2) suggested a lack of reaction, consistent with sensory deficits.

### vDISCO nanobody labelling and clearing

vDISCO was performed as previously described^2,45^ in combination with active pumping GFP-Nanobooster labelling (Atto647N-conjugated anti-GFP nanobooster Chromotek, gba647n-100) for 6 days and passive labelling for 3 days. This approach amplifies the endogenous eGFP signal in reporter mice and shifts it into the far-red spectrum, substantially improving signal-to-noise ratios throughout the tissue. Mice underwent DISCO clearing^46^ using a tetrahydrofuran (THF)/H_2_O series (50% THF, 70% THF twice, 90% THF, 100% THF) for 24 h per step followed by an incubation in dichloromethane for 6 h. Tissues were incubated in benzyl alcohol/benzyl benzoate (BABB, 1:2 (v/v)) until tissue transparency was reached (>48 h).

### WildDISCO antibody labelling and clearing

WildDISCO antibody labelling was performed as previously described in combination with anti-UCHL1 (14730-1-AP1, Proteintech, 26 µl per 200 ml immunostaining buffer) and anti-CGRP (ab36001, Abcam, 26 µl per 200 ml immunostaining buffer)^28^. Mice underwent DISCO clearing as described above.

### Fluorescence light-sheet imaging

Light-sheet imaging for whole-mouse bodies was conducted using a dipping ×1.1 objective lens (Miltenyi BioTec) on an Ultramicroscope Blaze (Miltenyi BioTec) using the ImspectorPro (v.5.1) software. Tiling scans (×1) were acquired using two-sided illumination with 35% overlap, 100% sheet-width, 0.1 NA, 100 ms exposure and a 6 µm *z*-step size. The images were taken in 16 bit depth and at a nominal resolution of 5.9 μm per voxel on the *xy* axes. Stitching of tile scans was carried out using Fiji’s stitching plugin with the ‘Stitch Sequence of Grids of Images’ feature^47^ and custom Python scripts. Imaging of mouse bodies at higher resolution was conducted using a ×4 objective lens (Miltenyi BioTec) on the same system as described above but tiling scans were acquired with the LightSpeed Mode using a 20% overlap, 80% sheet-width, 0.35 NA and 5 ms exposure time and a 6 µm *z*-step size. The images were taken in 16 bit depth and at a nominal resolution of 1.62 μm per voxel on the *xy* axes.

### 3D reconstruction

Dorsal and ventral scans were fused as previously described^2^ using Arivis (v.3.0.1 and v.3.4) and the exported whole-body TIFF stacks were used for image analysis.

### VR data annotation

Annotation of ground-truth data was performed in VR^7^ using the syGlass software (v.2.0.0) as previously described. To develop a robust and generalizable nerve segmentation model, a large and diverse dataset was curated from *Uchl1*-eGFP mouse scans imaged with the ×1.1 objective and annotated in VR. In total, the dataset comprised 1,217 patches (300 × 300 × 300 voxels) derived from 84 small subvolumes (300 × 300 × 300 voxels) and 8 larger subvolumes (~1,000 × 1,000 × 1,000 voxels). All large subvolumes were uniformly cropped into patches of 300 × 300 × 300 voxels to standardize the dataset. The training set incorporated 28 patches from *Uchl1*-eGFP volumes covering a range of anatomical contexts, 537 patches derived from 5 larger subvolumes of trigeminal nerves, and 118 patches from 1 larger subvolume of vertebral nerves. Together, these samples capture broad variations in nerve morphology and topological organization across the mouse body. To further enhance discriminative performance, particularly in regions susceptible to false-positive predictions, 29 negative sample patches containing structures such as adipocytes were included. For model evaluation, the testing set consisting of 7 patches from different parts of the mouse body, 478 trigeminal nerve patches cropped from 2 larger subvolumes of trigeminal nerves, 6 patches containing vertebral nerves and 14 negative patches. This design ensured thorough assessment of both segmentation accuracy and model generalizability across anatomical scales and tissue environments.

VR-annotation for *Cd68-*eGFP^+^ cells was performed in five 256 × 256 × 256 voxel patches from *Cd68*-eGFP whole-mouse scans, selected from representative regions of interest. Annotations were based on both the autofluorescence and *Cd68*-eGFP signal channels. These patches were further cropped down into 40 128 × 128 × 128 voxel patches that were used to train 3D networks for the segmentation of the markers of interest. Moreover, five 128 × 128 × 128 voxel patches were annotated as an independent test set used for evaluation.

For the development of the Tissue-Module, we annotated 27 organs of interest (Supplementary Table 7) in 12 downsampled (tenfold) mouse scans (6 from *Cd68*-eGFP and 6 from *Uchl1*-eGFP mice, with 6 chow-fed and 6 HFD-fed mice in total) using the autofluorescence and PI channels with the syGlass software. This approach was sufficient to distinguish all organs of interest. To generate reference annotations for the tissue segmentation, we annotated an initial dataset of three 1,024 × 1,024 × 1,024-voxel-sized patches in full resolution, containing 500 million voxels of fat (visceral, subcutaneous and brown), 145 million voxels of muscle, 16 million voxels of bone tissue and 8 million voxels of bone marrow. We iteratively increase the size of our annotated dataset through inference on unannotated patches, and manual correction of the wrongly segmented areas.

### Peripheral nerve segmentation

We developed the Nerve-Module of MouseMapper for nerve segmentation by fine-tuning a pretrained foundation model, VesselFM^14^, using our curated dataset (described above). VesselFM was pretrained on a large-scale 3D vessel dataset and was originally designed for the broad task of 3D blood vessel segmentation. To adapt VesselFM to our nerve dataset, we used an incremental learning strategy, learning without forgetting (LwF)^48^. Using learning without forgetting, the model was fine-tuned on nerve-specific data while regularizing its outputs to general vessel-related structural knowledge represented in the pretrained weights, thereby reducing the risk of catastrophic forgetting^49^. This approach allows the model to efficiently leverage prior knowledge while ensuring stable convergence on nerve data.

The fine-tuning process was implemented using a patch size of 128 × 128 × 128, an initial learning rate of 1 × 10^−3^ with scheduled decay, the stochastic gradient descent (SGD) optimizer and a segmentation loss combining Cross Entropy loss and Dice loss^50^. Incorporating learning without forgetting, each training batch obtains two sets of outputs: predictions for the nerve segmentation task from the fine-tuning model and ‘soft targets’ from the fixed pretrained VesselFM model representing the original vessel segmentation task. The final loss is computed as the sum of the nerve segmentation loss and a distillation loss, Kullback–Leibler divergence, that penalizes deviations from the pretrained model’s outputs. A weighting factor balances the segmentation and distillation losses, controlling the trade-off between retaining prior knowledge and learning nerve-specific features. In our experiments, optimal performance was achieved with a weighting factor of 0.4 for the distillation loss. The model was trained for 1,250 epochs.

Before forwarding the patches into the network for training or testing, we performed sample-wise normalization. Specifically, during the training, for each group of patches, including patches from whole-body, trigeminal nerve, vertebral nerve and negative samples, we computed the 0.5th percentile and 99.5th percentile of all voxel intensity values to set the minimum and maximum thresholds. Intensity values below or above these thresholds were clipped accordingly, followed by min–max normalization. During the testing, the same normalization procedure was applied to the entire testing dataset. This normalization step enhanced image contrast by stretching the intensity range between the chosen percentiles and removing outliers, thereby emphasizing nerve regions to improve model performance.

We compared our nerve segmentation model with other advanced 3D image segmentation networks^50^ (Supplementary Table 1): VNet^51^, Attention U-Net^52^, nnFormer^53^, UNETR^54^, SwinUNETR^55^, nnU-Net^56^ and nnUNetRes^56^. Each network was trained on the same training dataset until full convergence, defined as no decrease in the training loss for ten consecutive epochs. Moreover, the original VesselFM was also included in the comparison, to demonstrate the impact of finetuning. To assess the generalizability of MouseMapper across different labelling strategies and species, we applied it to diverse external datasets, including *Thy1-*eGFP vDISCO-labelled mice, wildDISCO antibody-labelled samples (tyrosine hydroxylase, UCHL1 and CGRP) and a publicly available post-conception-week 7 human embryo stained for β3-tubulin (https://hudeca.com). The human dataset is licensed under the Creative Commons Attribution-NonCommercial 4.0 International (CC BY-NC 4.0).

### Immune cell segmentation

For training the CD68 segmentation network (Immune-Module), we also fine-tune the VesselFM foundation model by freezing the encoder and finetuning the decoder. Thus, we can leverage the vast training of the foundation model by keeping its learned filters, but adapt the segmentation output through learning on our annotated dataset. As comparison baselines, we implemented the following architectures: 3D UNet^57^_,_ V-Net^51^, Attention U-Net^52^, nnFormer^53^ and UNETR^54^. All were trained by using the nnU-Net pipeline^56^, with a patch size of 128 × 128 × 128 voxels, channel-wise *z*-score normalization, learning rate decay and SGD optimizer. The baselines were trained until convergence for 1,000 epochs and initial learning rates of [0.0001, 0.001, 0.01], whereas the best performance for VesselFM was achieved by finetuning the network’s decoder for 500 epochs with an initial learning rate of 0.01. We train using fivefold cross validation, and evaluate voxel Dice, instance Dice^18^ and report best score per architecture. Based on these metrics, we selected the finetuned VesselFM for carrying out our downstream quantifications.

### Organ and tissue segmentation

For the segmentation of internal organs, we used eight annotated mice (from the *Cd68-*eGFP and *Uchl1-*eGFP line) to train five different networks: 3D UNet^57^, V-Net^51^, Attention U-Net^52^, nnFormer^53^ and Swin UNETR^55^. All of the architectures were trained through the nnU-Net pipeline^56^ using* z*-score normalization of each channel, and foreground oversampling. The networks were trained with SGD optimizer, using a batch size of 2, patch size of 64 × 256 × 128 voxels, initial learning rates of [1 × 10^−4^, 1 × 10^−3^, 1 × 10^−2^] and learning rate decay, for a total of 1,000 epochs. The resulting networks were evaluated on two *Cd68-*eGFP and two *Uchl1-*eGFP mouse reconstructions. During training, we performed fivefold cross validation, and the final predictions were made by ensembling the five resulting networks. We report best voxel Dice scores in Supplementary Table 7 for each architecture. We identified the 3D UNet as the best performing network layout, with the following properties: 6 downsampling layers, 6 upsampling layers, 3 × 3 × 3 sized convolutional blocks and a maximum feature size of 320 in the bottleneck, trained with the initial learning rate of 0.01.

Second, we train a model to segment the soft tissues of mice, such as muscle and adipose tissue. We iteratively increased the size of our annotated dataset through inference on unannotated patches, and manual correction of the wrongly segmented areas. As a result, our final networks were trained on a dataset of 387 samples containing a total volume of 2 billion voxels of adipose tissue and 2 billion voxels of muscle. We then train on these patches the following neural network architectures: 3D UNet, V-Net, Attention UNet and UNETR. We trained using fivefold cross-validation and, for evaluation, we report and select based on the validation scores of the ensembles of the five resulting networks (Supplementary Table 8). The networks were trained with SGD optimizer, using a batch size of 2, patch size of 128 × 128 × 128 voxels, initial learning rate of [1 × 10^−3^, 1 × 10^−2^] and learning rate decay, for a total of 1,000 epochs. The convolutional 3D UNet performs best among the implemented baselines.

The final inference pipeline of the Tissue-Module first segments the organs and then the tissues. First, the autofluorescence and PI channels of the acquired LSFM stack are downsampled to a resolution of 59 × 59 × 60 μm per voxel and saved as a 3D NIfTI volume. This is then fed into the organ segmentation network. The result is a 3D volume containing the masks of the 27 organs of interest, which can be used downstream for localizing structures of interest within organs, or for the quantification of organ volumes. Next, the organ masks are upsampled, and a non-organ mask is calculated, which is applied to the original scan. Through this process, we obtain a mask of the mouse volume that does not contain internal organs. This can be applied to the full-resolution scans, on which sliding-window inference can be carried out with the tissue segmentation model, as described below, to obtain the tissue map. Lastly, by combining the organ maps and the tissue maps, we obtain a spatial segmentation of major organs and tissues in the mouse body.

### Whole-body inference

To apply the nerve, immune and tissue segmentation network to whole-body scans in full resolution efficiently, we adapted the sliding window inference method previously used for segmentation tasks in medical images (MONAI)^58^ and the mouse brain (DELIVR)^7^. Our inference is implemented using the highly efficient ZARR file format and DASK parallel computing framework, enabling lazy loading and multiprocessing for data handling and writing tasks and, therefore, a rapid full-body analysis.

Before inference, we applied percentile normalization to each scan, similar to the model training stage. Given the significant imbalance between nerve/CD68^+^ voxels and background voxels in whole-body scans, we computed the 0.10th percentile and 99.9th percentile of all non-zero voxel intensity values to set the minimum and maximum thresholds, to effectively enhance the contrast between nerves and the background. During inference, we use the same patch normalization protocol as during network training, and patch size is selected to fit the memory resources available.

### *Cd68-*eGFP segmentation quantification

The binary masks obtained after CD68 marker segmentation were split into components by using the cc3d library^59^ for connected component analysis on subregions of the full-resolution scans. Subsequently, each individual detected connected component was post-processed by storing its location, volume, centre of mass, and shape^27^. Based on the location of the centre of mass, we automatically assign each segmented *Cd68-*eGFP^+^ cluster to the internal organs or the segmented tissues, with blobs not located in any of these being discarded as false positives. We further discard components of which the shape was elongated (string-like) as false positives, as these can often be artifacts, representing high-contrast blood vessels or nerves. Lastly, we grouped the detected *Cd68-*eGFP clusters into three discrete categories, based on their volume (amount of segmented voxels within a component): small (smaller than 50 voxels), medium (between 50 and 500 voxels) and large (over 500 voxels). We chose these categories based on the observation that, when considering the total spatial volume of all clusters, each of these three categories would represent a similar amount (approximately 30%) of the total CD68^+^ segmented volume. Then, for each mouse and for each organ or tissue, we studied the percentage composition of each of these categories, and analysed the differences between the chow and HFD groups.

While applying the CD68 segmentation network to the whole-mouse bodies, we observed that it displays zero-shot transfer learning abilities in the limited setting of applying the model in inference to certain novel tissues, where we observed positive detections. Thus, to validate any reported changes, we performed (1) visual analysis of the resulting segmentation, and (2) a VR-based annotation of a representative test patch in the tissue of interest. We compared the result of the automatic segmentation against the manual annotation in order to evaluate the network’s transfer learning abilities. We only considered valid quantifications where the network passed with a Dice score >60%.

### *Uchl1-*eGFP segmentation quantification

After inference, we obtained the whole-body nerve segmentation of *Uchl1-*eGFP mice. We then performed connected component analysis to post-process the segmentation results, eliminating large false-positive segments caused by high-intensity regions within the mouse body. Subsequently, we quantified the nerve voxels and density from three perspectives: the entire body, individual tissues and specific organs.

To quantify nerves in the entire body, the organ and tissue segmentations from the Tissue-Module were combined to form a binary mask of major organs and tissues in the mouse body. By dilating this binary mask, we created a whole-body mask that covers the entire mouse body, enabling us to compute the nerve voxels and density within. For tissue-wise quantification, the tissue segmentation from the Tissue-Module was used to calculate the nerve voxels and density in fat and muscle tissues. To further distinguish adipose compartments, we manually separated the visceral and subcutaneous fat within the tissue mask by referencing the abdominal wall mask derived from organ segmentation network. For quantifying nerves for specific organs, we accounted for structures in the immediate vicinity of the organs by extending the organ segmentation by 500 µm to calculate the organ wise statistics. Notably, to create the head mask, we overlaid the dilated brain masked with whole-body mask followed by minor manual refinement, resulting in a precise mask for quantifying the nerve voxels inside. For the 4× scan analysis, we included the limbs as regions of interest. Using the whole-body mask and referencing the head and heart masks as anatomical landmarks, we defined an initial limb region, which is located lateral to the head and superior to the top of the heart. This preliminary region was then manually refined to generate the final limb mask used for nerve voxel and density analysis.

### Graph extraction

Graph extraction was performed as previously described^21,60^. Similarly we extracted the skeletonization, depth map and extracted a graph of the resulting skeleton. All small, isolated subgraphs were pruned from the graph. As the resulting image data were too large to fit into a reasonable amount of RAM, we separated the whole image into sub-blocks using nibabel. We next extracted the graphs from each sub-block and merged them together. We fused all nodes together on the border between two blocks where the Euclidian distance between nodes was less than a given threshold by introducing a new edge between the nodes. We quantify the thickness of each node and each edge using the depth map, the degree of each node and the number of leaf nodes (nodes with degree = 1).

### Computational load of MouseMapper

The experiments presented in this work were carried out using a cohort of 19 mice (10 HFD fed, 9 chow fed). The nine 1× *Uchl1*-eGFP whole-body scans (4 chow and 5 HFD) generated a total of 105,948 2D *z*-slices and 10.926 trillion voxels, occupying 9.42 TB after ZARR compression. Moreover, nine 4× UCHL1 ventral scans from the same mice, together with two 4× dorsal scans from two of them (1 chow and 1 HFD) produced another 64,968 2D *z*-slices and 54.888 trillion voxels, occupying 56.1 TB after compression. The ten 1× *Cd68*-eGFP whole-body scans of 5 chow-fed and 5 HFD-fed mice generated a total of 112,515 *z*-slices and 10.779 trillion voxels, occupying 7.48 TB after compression. Two additional 4× *Cd68*-eGFP ventral scans from two mice (1 chow fed and 1 HFD fed) produced another 11,706 2D *z*-slices and 9.726 trillion voxels, occupying 9.8 TB after compression. To accurately quantify these data, our annotation efforts resulted in significantly ample datasets. For the Nerve-Module, we manually annotated 72 GB of data. While building the Immune-Module, we annotated 350 MB of data from representative areas in visceral and subcutaneous fat, as well as in the peritoneum. The organ segmentation network of the Tissue Module was trained using 10 GB of downsampled organ data, whereas the tissue segmentation network (for fat, muscle, bone and bone marrow) was trained using 46 GB of full-resolution tissue annotations, built as a mixture of manual and automatic annotations. To train the networks building our MouseMapper pipeline, as well as to run the predictions and quantifications presented in this paper, the High-Performance Computing cluster of Helmholtz Zentrum Munich was used. Thus, the processes could be parallelized and carried out more efficiently.

We estimate approximately 500 GPU hours for multiple model training and evaluation, 265 GPU hours for 1× segmentations of scans (nerves, CD68^+^ cells, tissues), about 550 GPU hours per 4× scan and 0.1 GPU hours for organ inference per scan. For nerve voxel and density calculations, we estimate roughly 330 CPU hours for all 1× scans and about 60 CPU hours per 4× scan. CD68^+^ blob post-processing used approximately 100 CPU-hours for all 1× scans. Graph extraction and postprocessing is performed solely on CPU. We estimate 216 CPU hours for 1× graph extraction.

### Spatial proteomics sample preparation

For spatial proteomics of trigeminal ganglia of *Uchl1-*eGFP mice, 18G needle punches were prepared from rehydrated trigeminal ganglia and subsequently used for proteomics sample preparations as described previously^11^. In brief, the samples were resuspended in 6% SDS buffer, heat denatured at 95 °C for 45 min at 600 rpm in a thermoshaker, sonicated in high mode for 30 cycles (30 s off, 30 s on) (Bioruptor Plus, Diagenode) and then precipitated using 80% acetone overnight at −20 °C. The next day, these samples were centrifuged and the pellet was resuspended in SDC lysis buffer (2% SDC, 100 mM Tris-HCl pH 8.5). The samples in the SDC buffer were sonicated in high mode for 15 cycles (30 s off, 30 s on) (Bioruptor Plus, Diagenode). The samples were again heated to 95 °C at 600 rpm in a thermoshaker for 45 min. The protein samples were digested overnight with trypsin and LysC (1:50, protease:protein ratio) at 37 °C with 1,000 rpm shaking. The resulting peptides were acidified with 1% trifluoroacetic acid (TFA)/99% isopropanol at a 1:1 volume-to-volume ratio, vortexed and centrifuged to pellet residual particles. The supernatant was transferred to fresh tubes and subjected to an in-house built StageTip clean-up consisting of three layers of styrene divinylbenzene reversed-phase sulfonate (SDB-RPS; 3 M Empore) membranes. Peptides were loaded onto the activated (100% acetonitrile, 1% TFA in 30% methanol and 0.2% TFA, respectively) StageTips, run through the SDB-RPS membranes and washed by ethyl acetate including 1% TFA, isopropanol including 1% TFA and 0.2% TFA, respectively. Peptides were then eluted from the membranes through 60 µl elution buffer (80% acetonitrile, 1.25% NH_4_OH) and dried using a vacuum centrifuge (40 min at 45 °C). Finally, peptides were reconstituted in 8–10 µl of loading buffer (2% acetonitrile, 0.1% TFA) and stored at −80 °C until further use.

For proteomics profiling of human trigeminal ganglia, samples were reduced and denatured in lysis buffer (2% SDC, 10 mM TCEP and 100 mM Tris-HCl pH 8.5, 40 mM chloroacetamide) at 95 °C for 45 min in a PCR thermocyler, sonicated in high mode for 30 cycles (30 s off, 30 s on; Bioruptor Plus; Diagenode) and heated again to 95 °C for 45 min. Contaminants and detergents were removed using SP3-based precipitation and washing on 5 µl magnetic beads^61^. In brief, 100 µl ethanol was used for precipitation, 50 µl ethanol was used for washing and proteins were then dried in the SpeedVac before adding trypsin and LysC proteases. A second overnight digestion step was added to improve digestion efficiency as previously described^11^.

### Evotip PURE clean-up of human samples

A total of 1 µg of each tissue digest was desalted per Evotip. The Evotip PURE protocol was adjusted for offline C18 clean-up in a 96-well format as described previously^10^. Initially, Evotip PURE tips were rinsed with 20 µl of buffer B (comprising 80% acetonitrile, water and 0.1% formic acid) and spun down at 800*g* for 60 s. The Evotips were conditioned with 10 µl of isopropanol, followed by a 1 min centrifugation at 100*g* and additional 1 min at 400*g* to empty the Evotips. The PURE Evotips were washed and equilibrated in 200 µl of buffer A (0.1% formic acid). The samples were acidified in 5% TFA, and the Evotip PURE was emptied by centrifuging at 800*g* for 1 min. The acidified samples were loaded onto the PURE Evotips and centrifuged at 800*g* for 1 min. The samples were washed with 200 µl of buffer A and spun down at 800*g* for 1 min. Elutions were collected in PCR strips by eluting with 20 µl of buffer B by centrifuging at 100*g* for 1 min followed by 450*g* 1 min. The peptides were dried in a SpeedVac and resuspended in 40 μl of 0.1% TFA supplemented with 0.015% DDM for MS analyses. Up to 2 μl (or 50 ng peptides) was injected per MS analysis.

### LC–MS

The MS data for mouse samples was acquired in data-independent acquisition (DIA) mode. The liquid chromatography–tandem mass spectrometry (LC–MS/MS) analysis was carried out using the EASY nanoLC 1200 (Thermo Fisher Scientific) system coupled to a trapped ion mobility spectrometry quadrupole time-of-flight single-cell proteomics mass spectrometer (timsTOF SCP, Bruker Daltonik) through a CaptiveSpray nano-electrospray ion source. Peptides (50 ng) were loaded onto a 25 cm Aurora Series UHPLC column with CaptiveSpray insert (75 μm inner diameter, 1.6 μm C18) at 50 °C and separated using a 50 min gradient (5–20% buffer B in 30 min, 20–29% buffer B in 9 min, 29–45% in 6 min, 45–95% in 5 min, wash with 95% buffer B for 5 min, 95–5% buffer B in 5 min) at a flow rate of 300 nl min^−1^. Buffers A and B were water with 0.1 vol% formic acid and 80:20:0.1 vol% acetonitrile:water:formic acid, respectively. MS data were acquired in single-shot library- free DIA mode and the timsTOF SCP was operated in DIA/parallel accumulation serial fragmentation (PASEF) using the high-sensitivity detection–low sample amount mode. The ion accumulation and ramp time were set to 100 ms each to achieve nearly 100% duty cycle. The collision energy was ramped linearly as a function of the mobility from 59 eV at 1/*K*_0_ = 1.6 Vs cm^−2^ to 20 eV at 1/*K*_0_ = 0.6 Vs cm^−2^. The isolation windows were defined as 24 × 25 Th from *m*/*z* 400 to 1,000. The MS data for human samples were acquired using similar as previously described using a 5.5 cm long mPAC HT column to minimize carryover and to accelerate column cleaning and maintenance between different sample types^10^.

### Proteomics data processing

For mouse data, diaPASEF raw files were searched against the mouse uniport database using DIA-NN^62^. Peptides length range from seven amino acids were considered for the search including N-terminal acetylation. Oxidation of methionine was set as a variable modification and cysteine carbamidomethylation as fixed modification. Enzyme specificity was set to trypsin/P with 2 missed cleavages. The FASTA digest for library-free search was enabled for predicting the library generation. The FDR was set to 1% at precursor and global protein level. The Match between runs feature was enabled and quantification mode was set to Robust LC (high precision). The protein group column in DIA-NN’s report was used to identify the protein group and PG.MaxLFQ was used to calculate the differential expression. For human data, analogous searches using DIANN v.2.0 were performed.

### Proteomics data analysis

Mouse data were analysed using scanpy (v.1.10.1) and anndata (v.0.8.0) in Python v.3.10. In total, 12 independent samples were analysed from each group (high-fat diet and chow) from three animals with samples from both right and left trigeminal ganglia. All proteins expressed in less than half of the samples in each group were filtered out, resulting in 6,686 proteins used for downstream analyses. The data were log-transformed and normalized per sample. The missing values were input using KNNImputer (n_neighbors=5) from the sklearn package (v.1.2.1). Using scanpy’s dendrogram function, scipy’s hierarchical linkage clustering was calculated on a Pearson correlation matrix over groups which was calculated for 50 averaged principal components. To identify differentially regulated proteins across two groups (HFD and chow), we combined samples from the right and the left trigeminal ganglia. Differential expression analysis was conducted using Scanpy’s method ‘rank_genes_groups’ with method set to ‘t-test’. We applied a threshold of *P* < 0.05 and |log[fold change]| > 0.5 to identify differentially expressed proteins (Supplementary Table 13). These differentially expressed proteins were subsequently visualized using volcano plots. Pathway enrichment analysis was performed on the combined upregulated and downregulated proteins using the KEGG and Reactome databases. The most relevant pathways were highlighted, displaying the differentially expressed proteins involved in each pathway. For human data, the significance of differences in protein abundances between the obese and lean groups was determined using Excel (v.2016) with a two-tailed Student’s *t*-test, applied to proteins that were identified in both groups at least three times. A FC threshold was set to proteins that were identified with significant changes: a decrease in abundance (log_2_[FC] < −0.5) or an increase in abundance (log_2_[FC] > 0.5), with a significance cut-off of *P* < 0.05. Significantly altered protein groups were subsequently searched in the KEGG and Reactome databases using the DAVID annotation tool (v.Dec. 2021, Knowledgebase v.2023q4). Pathway analyses were performed by filtering for significant pathways (*P* < 0.05; Supplementary Table 12). A subset of pathways displayed in Fig. 4h were manually selected for visualization using Python (v.3.8).

### Western blot

Protein lysates from trigeminal ganglia were prepared by homogenizing frozen tissue in RIPA buffer with freshly added inhibitors (1× EDTA-free protease inhibitor and 1× PhosSTOP) using the Tissuelyzer II (Qiagen). The samples were centrifuged at 13,000*g* and 4 °C for 30 min. The protein content of cleared lysates was determined using the Pierce BCA Kit (Thermo Fisher Scientific, 23225). Protein lysates were incubated with 6× Laemmli buffer at 95 °C for 5 min before loading it onto an SDS–PAGE gel (Novex WedgeWell, Tris-Glycine Mini Gels; Thermo Fisher Scientific, or Mini-PROTEAN Precast Gels; Bio-Rad Laboratories). Gels were run at 100–120 V and subsequently transferred to a nitrocellulose membrane (Bio-Rad). The membranes were blocked with 5% skimmed milk in TBS-T and incubated with primary antibodies against SEPTIN7 (Proteintech, 13818-1-AP), SERPINA1 (Proteintech, 16382-1-AP, p-ERK (Cell Signaling, phospho-p44/42 MAPK (Thr202/Tyr204), 9101) ERK (p44/42 MAPK, 9102) diluted 1:1,000 in 5% BSA V5. Vinculin (EPR8185, Abcam, ab129002) was diluted 1:10,000 in 5% BSA V5. Anti-rabbit IgG coupled to horseradish peroxidase were used at a dilution of 1:10,000 in 5% milk as secondary antibodies, and immunoreactive proteins were determined by chemiluminescence using the ChemiDoc MP System (Bio-Rad).

### Multiplex antibody labelling and analysis

The MACSima Imaging Cyclic Staining technology from Miltenyi Biotec was performed according to the manufacturer’s protocol on paraffin-embedded tissue sections of epididymal white adipose tissue of obese mice^63^. Image acquisition was performed automatically by the MACSima instrument in seven regions of interest (ROIs). The following antibodies from Milteny Biotech were used at a dilution of 1:10: NK1.1 (REA1162), CD3 (REA641), F4/80 (REA126), MHC-II (REA813), CD31 (REAL260) and CD138 (REA104). Images were thresholded individually for each marker to optimize visualization and analysed using the spatiomic package^64^ (v.0.8.0). For each ROI a 30 × 30 self-organizing map (SOM) was trained on a subset of 1 million pixels. The SOM compresses the pixel data into a smaller set of representative prototypes, which were subsequently clustered using the Leiden algorithm^65^. The Leiden clusters were mapped to each pixel according to their SOM mapping. The clusters were annotated with cell types based on mean intensities in each cluster. The vicinity cluster compositions for each cluster were retrieved per ROI using spatiomic’s ‘vicinity_composition’ function. The ROIs were combined by summing the compositions of each ROI. The vicinity graph was retrieved from these combined compositions.

### Statistical analysis

Results from biological replicates were expressed as mean ± s.e.m. Statistical analysis was performed using GraphPad Prism (v.9). To compare two conditions, unpaired Student’s* t*-tests or Mann–Whitney *U*-tests were performed. Insulin-tolerance tests were analysed using two-way ANOVA with Šídák’s multiple-comparison test. Proteomics data analysis was performed as described above. No statistical method was used to predetermine sample size. Mice were randomly assigned to chow or HFD groups. Unless stated otherwise, investigators were not blinded during experiments or data analysis.

### Reporting summary

Further information on research design is available in the Nature Portfolio Reporting Summary linked to this article.

## Online content

Any methods, additional references, Nature Portfolio reporting summaries, source data, extended data, supplementary information, acknowledgements, peer review information; details of author contributions and competing interests; and statements of data and code availability are available at 10.1038/s41586-026-10535-2.

## Supplementary information

Supplementary Information Supplementary Figs. 1–6 and Supplementary Tables 1–11.

Reporting Summary

Supplementary Table 12 Proteomic profiling and pathway enrichment analysis of human trigeminal ganglia.

Supplementary Table 13 Differentially expressed proteins in mouse trigeminal ganglia.

Supplementary Video 1 Whole-body reconstructions of normal (left) and obese (right) UCHL1–eGFP mice. Peripheral nerves (*Uchl1*-eGFP^+^) are shown in green; bones and organs are shown in red (PI labelled) and muscle (autofluorescence) is shown in blue.

Supplementary Video 2 Whole-body reconstructions of normal (left) and obese (right) CD68–eGFP mice. Immune cells (*Cd68-*eGFP^+^) are shown in cyan; bones and organs are shown in magenta (PI labelled) and muscle (autofluorescence) is shown in yellow.

Supplementary Video 3 Whole-body reconstructions of a lean UCHL1–eGFP mouse showing *Uchl1*-eGFP^+^ nerves (green) in the whole mouse. Bones and organs are shown in red (PI labelled) and muscle (autofluorescence) is shown in white.

Supplementary Video 4 Whole-body reconstructions of an obese UCHL1–eGFP mouse showing *Uchl1*-eGFP^+^ nerves (green) in the whole mouse. Bones and organs are shown in red (PI labelled) and muscle (autofluorescence) is shown in white.

Supplementary Video 5 Whole-body reconstructions of a chow-fed CD68–eGFP mouse showing *Cd68-*eGFP^+^ cells in the whole mouse. Immune cells (*Cd68-*eGFP^+^) are shown in cyan, bones and organs are shown in magenta (PI labelled) and muscle (autofluorescence) is shown in yellow.

Supplementary Video 6 Whole-body reconstructions of a HFD-fed CD68–eGFP mouse showing infiltrating *Cd68-*eGFP^+^ cells in the whole mouse with accumulations in ScAT, ViscAT and the peritoneum. Immune cells (*Cd68-*eGFP^+^) are shown in cyan, bones and organs are shown in magenta (PI labelled) and muscle (autofluorescence) is shown in yellow.

Supplementary Video 7 Higher-resolution CD68–eGFP imaging (×4, 0.35 NA) enables visualization of single cells within different tissues.

Supplementary Video 8 Overlay of UCHL1–eGFP with the AI-based segmentation generated by MouseMapper in limbs and adipose tissue.

Supplementary Video 9 Representative chow-fed mouse showing AI-segmented organs and tissue. Each colour represents a different organ or tissue segmented using the Tissue-Module of MouseMapper.

Supplementary Video 10 Representative obese mouse showing AI-segmented organs and tissue. Each colour represents a different organ or tissue segmented using the Tissue-Module of MouseMapper.

Supplementary Video 11 Tissue-Module organ and tissue segmentation with progressive structural reveal. The video first shows the whole mouse body with all segmented organs and tissues. Next, muscle and adipose tissues are stripped away, revealing only the organ structures displayed in a mesh-based 3D view. Finally, the corresponding raw imaging data are shown to illustrate the anatomical accuracy of the segmented structures.

Supplementary Video 12 Representative obese CD68–eGFP mouse showing AI-segmented immune cell clusters (blue, small-sized clusters; green, medium-sized clusters; red, large-sized clusters).

## Source data

Source Data Fig. 3

Source Data Fig. 4

Source Data Fig. 5

Source Data Extended Data Fig. 1

Source Data Extended Data Fig. 5

Source Data Extended Data Fig. 7

Source Data Extended Data Fig. 8

Source Data Extended Data Fig. 9

## Data Availability

Links to whole-body scans are available online (https://discotechnologies.org/MouseMapper/). MS raw data libraries and outputs from the search engine have been deposited to the ProteomeXchange Consortium through the PRIDE partner repository: mouse trigeminal ganglion data (PXD054819); human trigeminal ganglion data (PXD071172). Source data are provided with this paper.

## Code availability

Our code is available at GitHub (https://github.com/erturklab/mouseMapper).
