# Supplementary material for: A deep-learning framework reveals whole-body perturbations at cell level
Source: Nature. 2026 May 20;655(8124):1016–26. doi: 10.1038/s41586-026-10535-2 (PMC13391364; doi:10.1038/s41586-026-10535-2)
Supplement: Supplementary file 1 — Supplementary Figs. 1–6 and Supplementary Tables 1–11. [file 41586_2026_10535_MOESM1_ESM.pdf]

nature portfolio

https://doi.org/10.1038/s41586-026-10535-2

Supplementary information

A deep-learning framework reveals whole-body perturbations at cell level

In the format provided by the authors and unedited

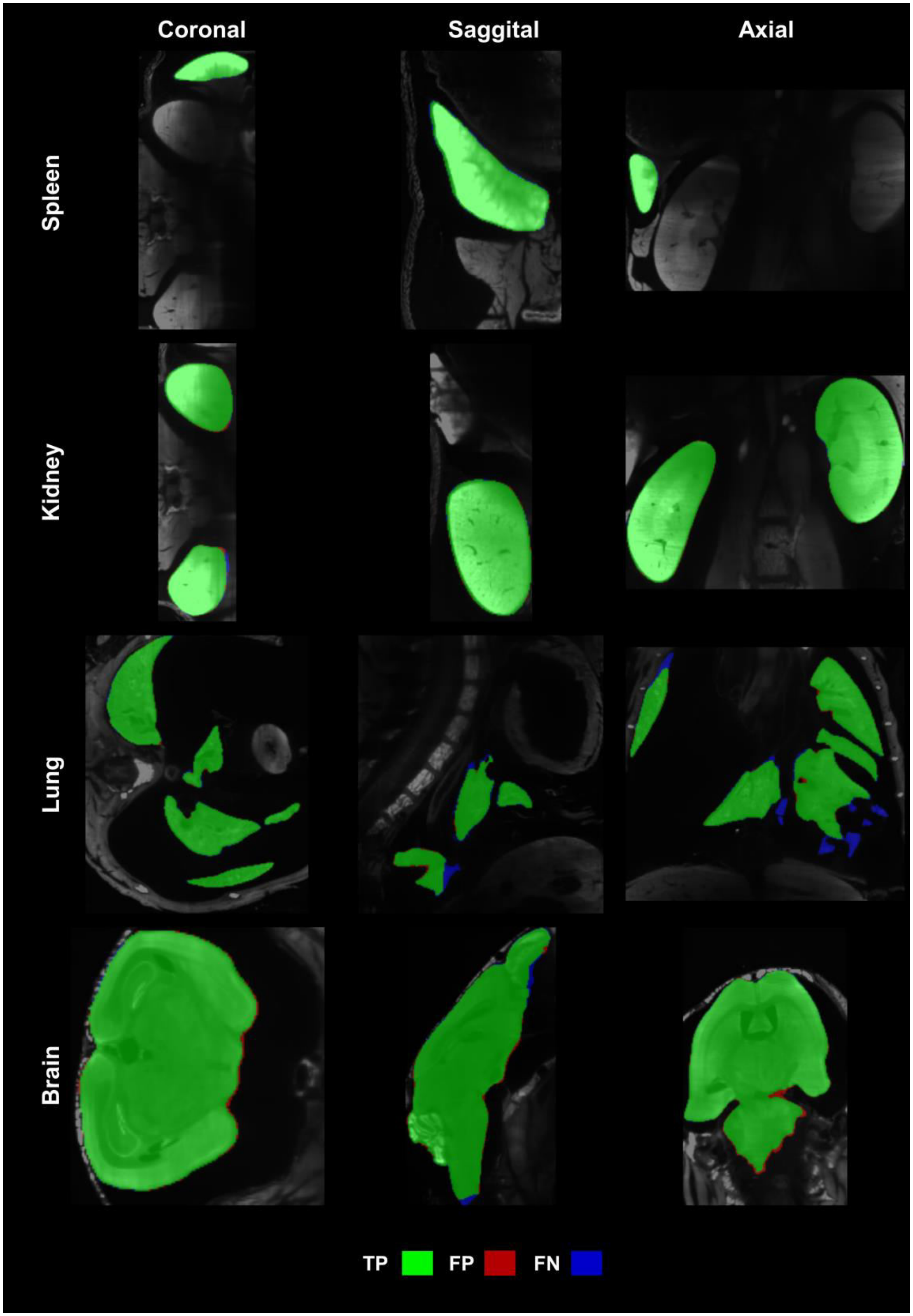

Supplementary Fig. 1: Evaluation of the network performance for the segmentation of indicated tissues based on volumetric Dice. Areas overlapping with reference: True positives (TP); no overlap in reference: false positives (FP); undetected reference: false negatives (FN).

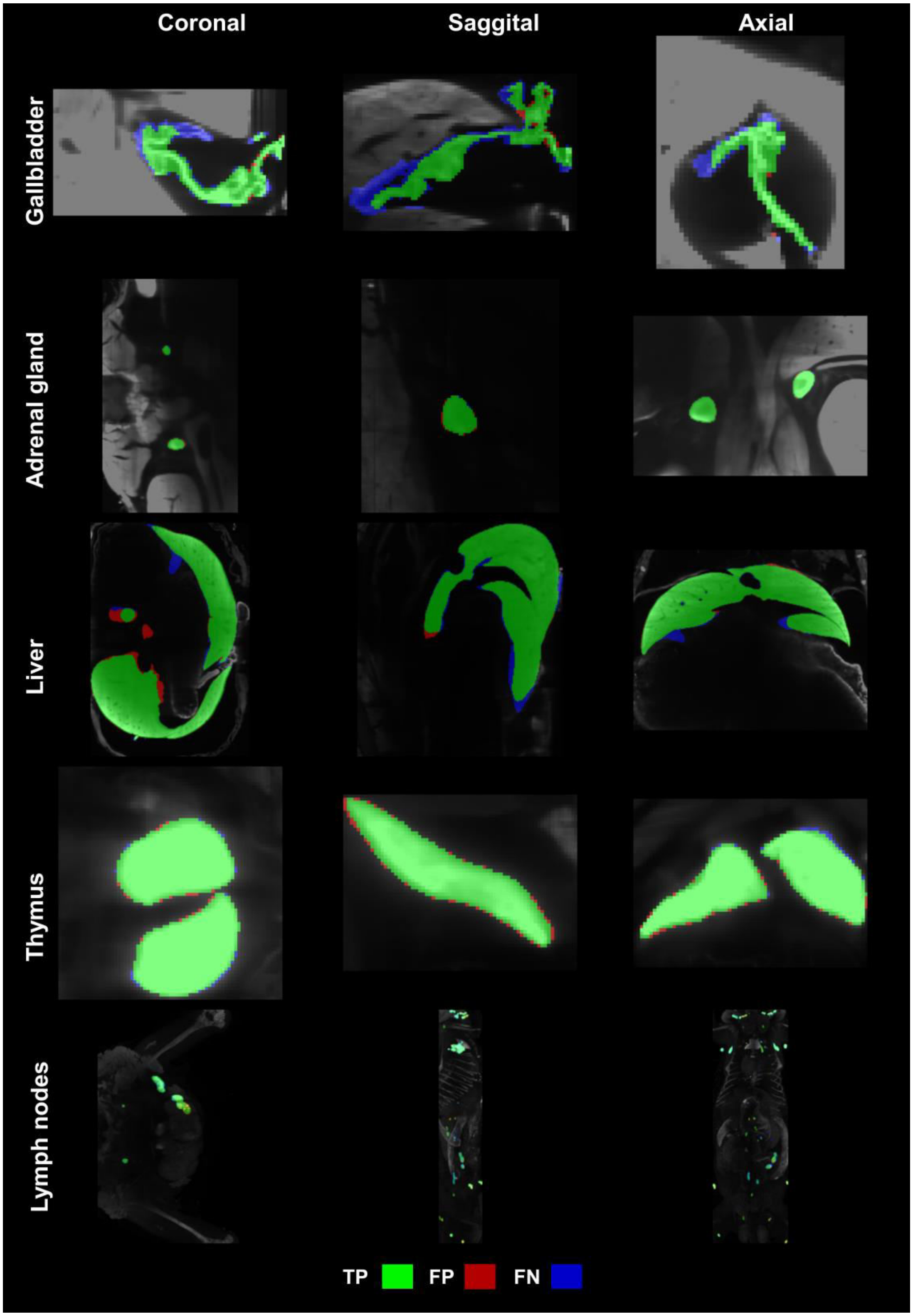

Supplementary Fig. 2: Evaluation of the network performance for the segmentation of indicated tissues based on volumetric Dice. Areas overlapping with reference: True positives (TP); no overlap in reference: false positives (FP); undetected reference: false negatives (FN).

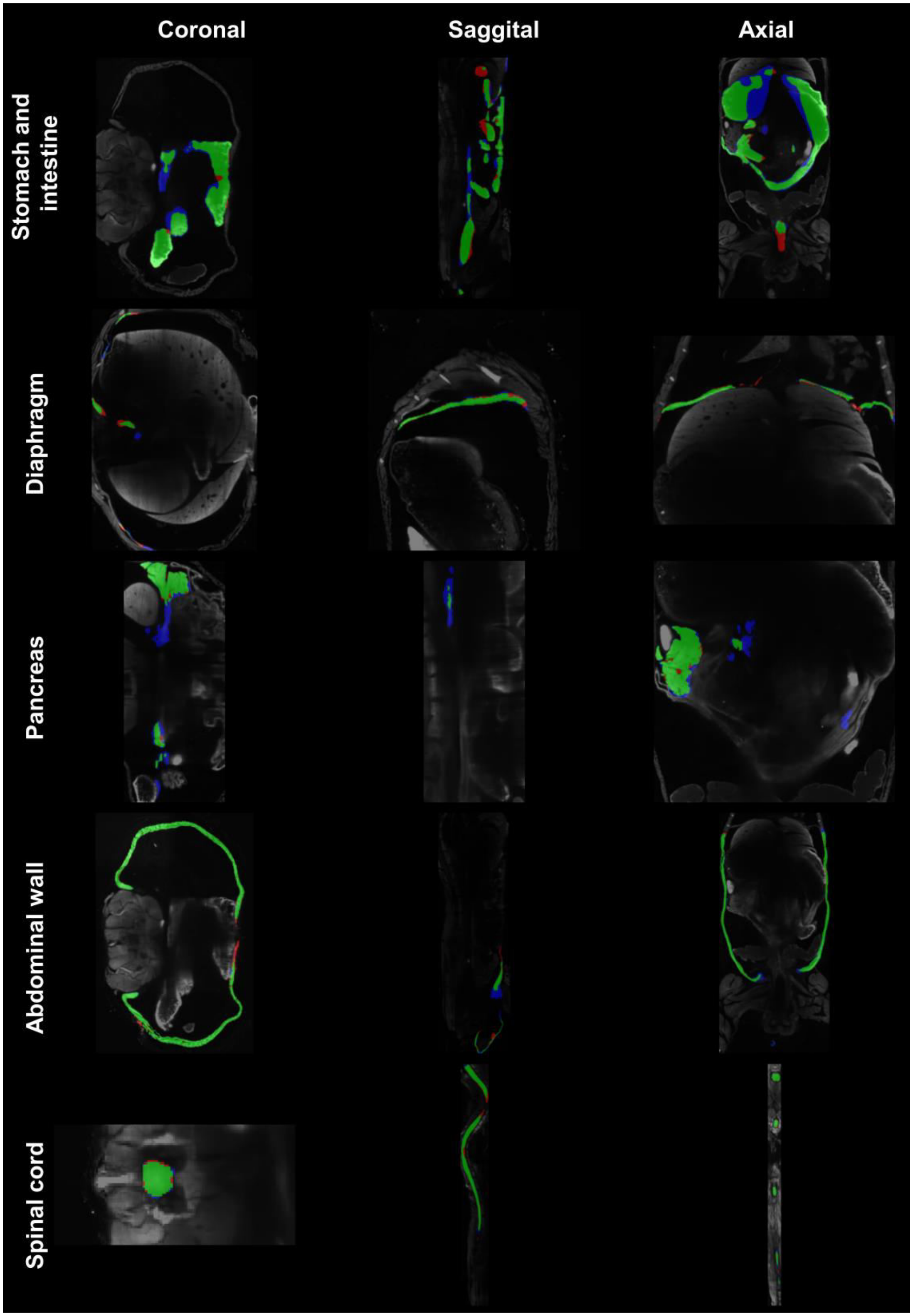

Supplementary Fig. 3: Evaluation of the network performance for the segmentation of indicated tissues based on volumetric Dice. Areas overlapping with reference: True positives (TP); no overlap in reference: false positives (FP); undetected reference: false negatives (FN).

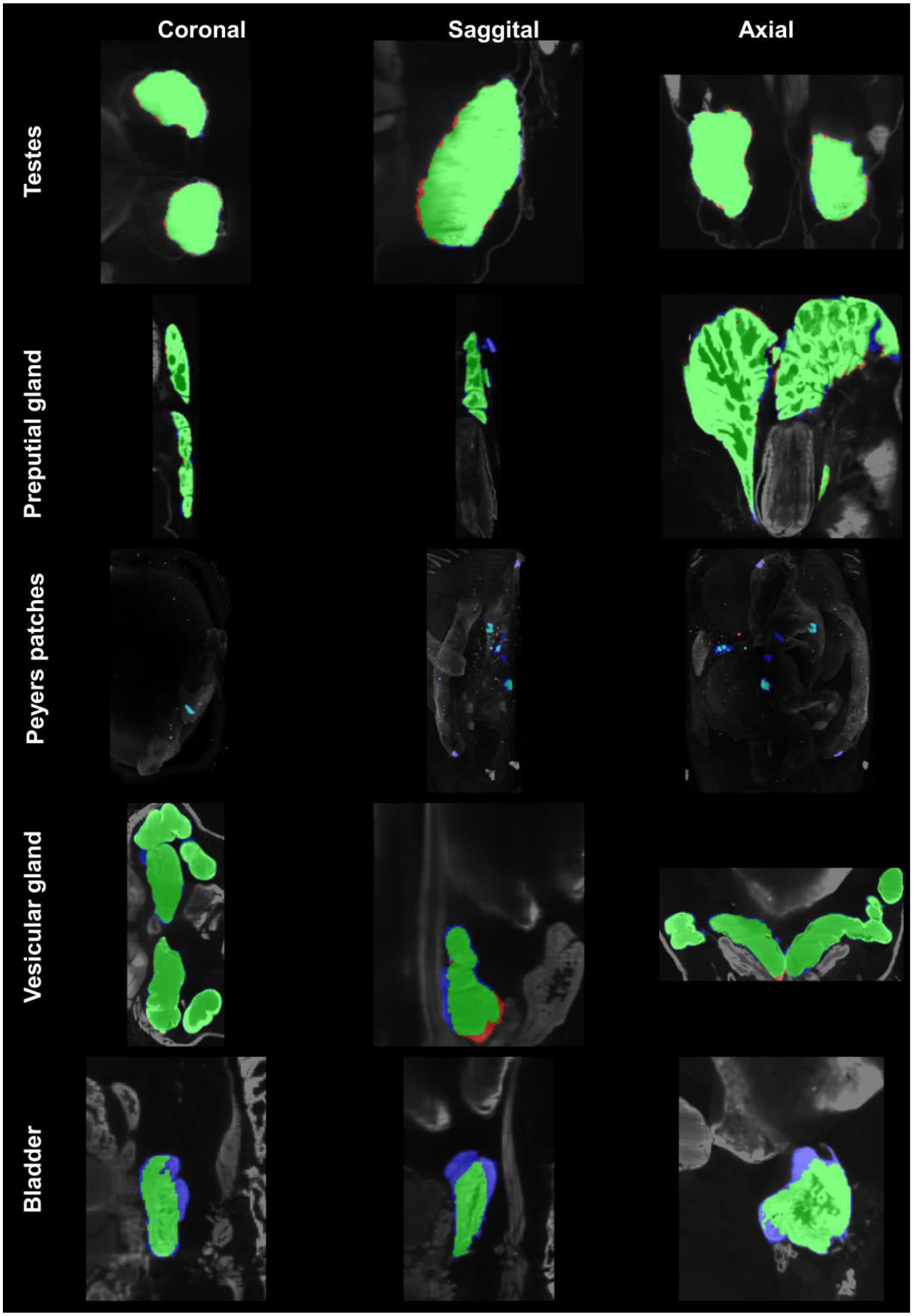

Supplementary Fig. 4: Evaluation of the network performance for the segmentation of indicated tissues based on volumetric Dice. Areas overlapping with reference: True positives (TP); no overlap in reference: false positives (FP); undetected reference: false negatives (FN).

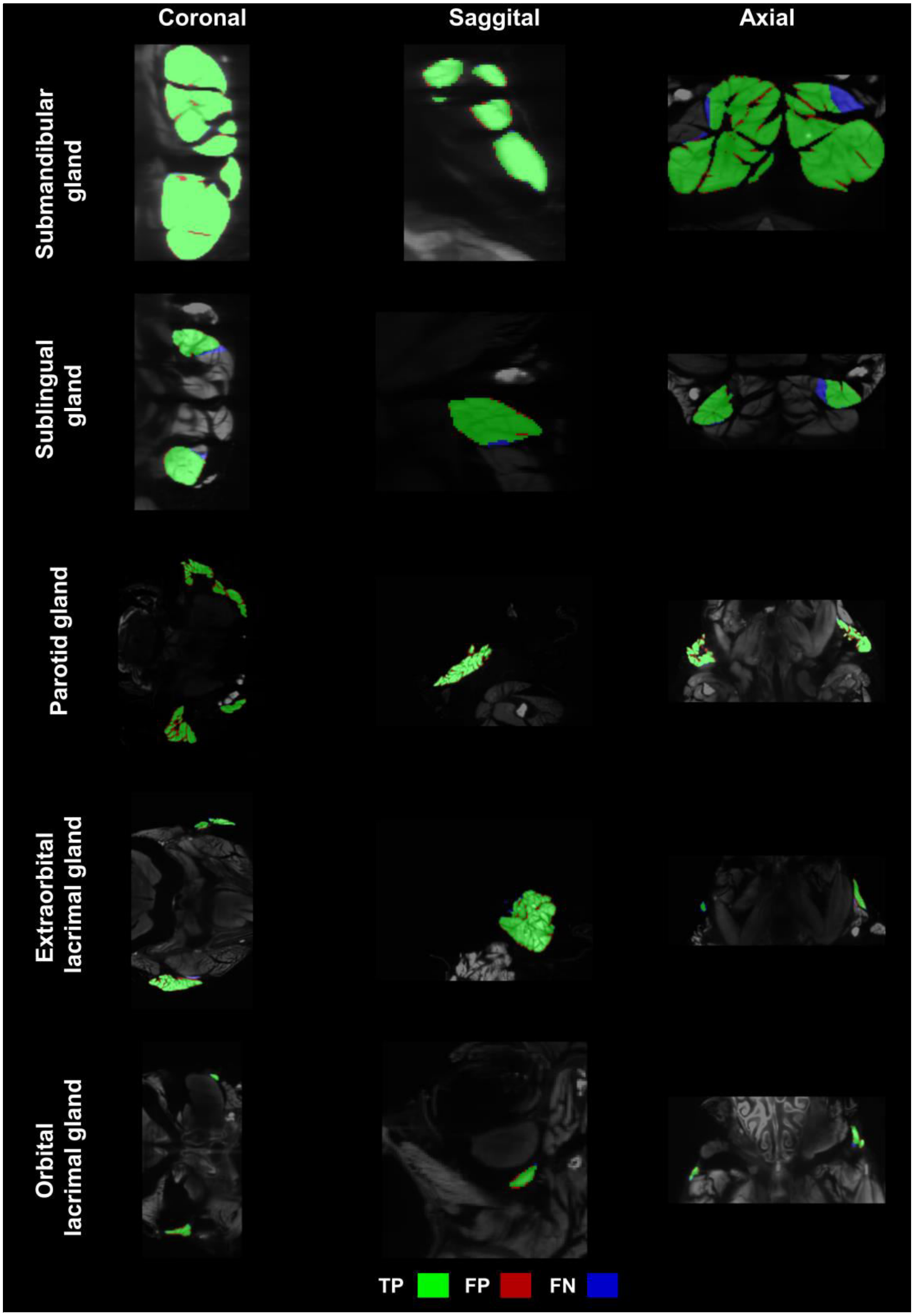

Supplementary Fig. 5: Evaluation of the network performance for the segmentation of indicated tissues based on volumetric Dice. Areas overlapping with reference: True positives (TP); no overlap in reference: false positives (FP); undetected reference: false negatives (FN).

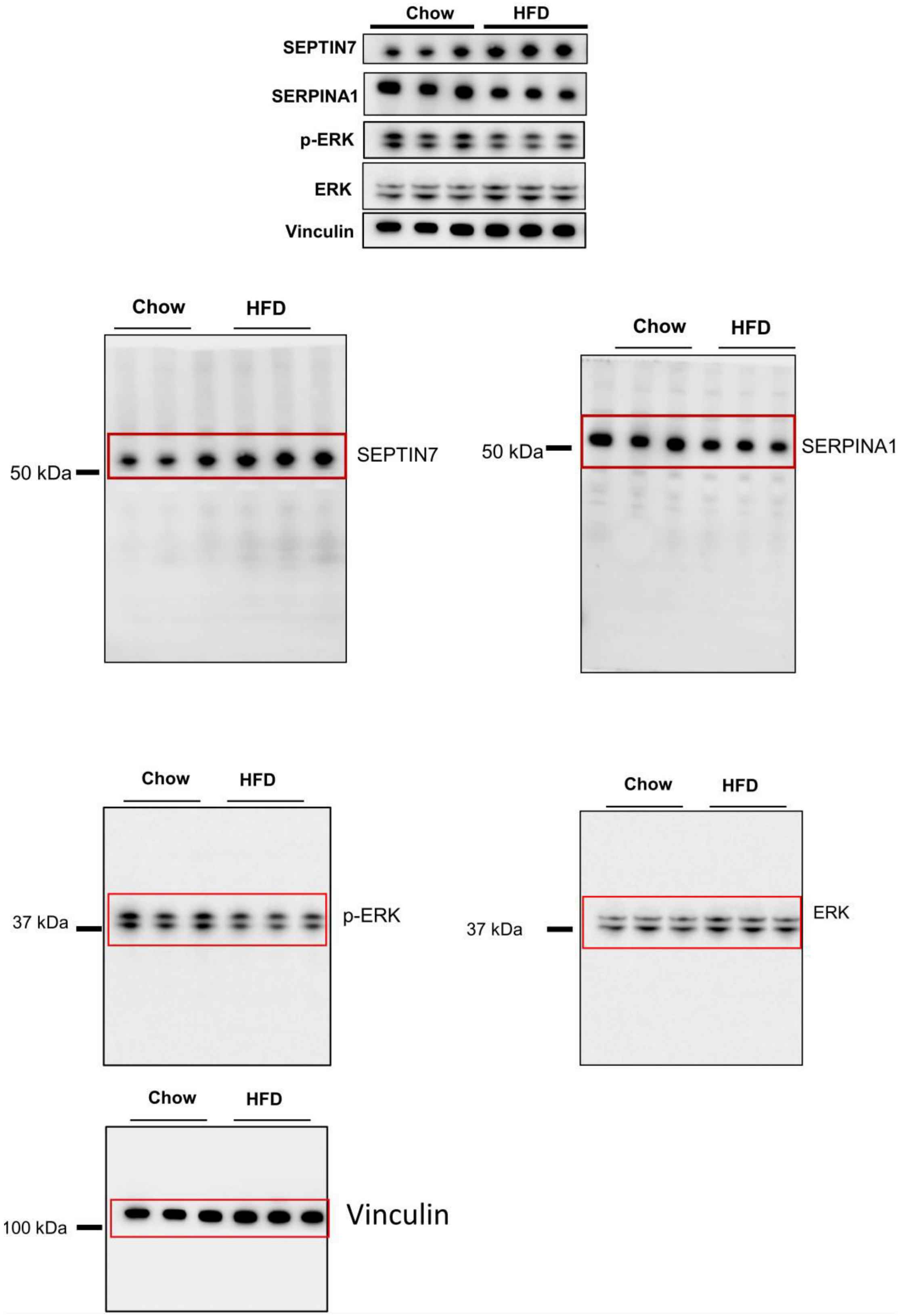

Supplementary Fig. 6: Western blot raw data of Extended Data Figure 8d (shown again in the top panel).

Supplementary Table 1: Evaluation of different networks for nerve segmentation based on the volumetric and centerline dice. The best performing scores are highlighted in bold. Our final model (MouseMapper) was developed by finetuning a foundation model.

	nnUNet Res	Atten-tion Unet	Nn-Former	Swin UNETR	UNET R	VNet	nn-UNet	nn-UNET with clDICE loss*	Vessel FM	**Mouse Mapper**
**Voxel Dice (%)**	72.35	70.33	74.22	62.78	66.59	62.78	70.81	72.03	19.06	**74.94**
**Centerl ine Dice (%)**	62.84	61.55	64.71	50.69	50.08	51.34	62.90	63.97	11.06	**66.64**

Supplementary Table 2: Benchmarking MouseMapper (finetuned foundation model) and different networks for nerve segmentation based on the voxel and centerline Dice on different nerve data sets, including increased magnification (UCHL1 4x), wildDisco antibody labelling (PGP9.5 and TH) and a different nerve reporter line (Thy1-EGFP).

		AttentionUnet	nnFormer	SwinUNETR	UNETR	VNet	nn-UNet	nnUNet Res	VesselFM	Mouse Mapper
UCHL1-EGFP 4x	Voxel Dice	69.05%	59.30%	56.70%	53.52%	59.14%	64.25%	55.25%	10.92%	69.16%
	Centerline Dice	49.72%	33.76%	33.90%	31.73%	45.18%	35.60%	14.77%	11.65%	50.00%
PGP9.5 4x (wild DISCO)	Voxel Dice	66.51%	71.02%	71.50%	47.69%	64.02%	59.43%	66.98%	45.18%	71.88%
	Centerline Dice	56.92%	62.25%	58.29%	43.97%	56.33%	53.11%	58.28%	37.20%	63.01%
TH Gut nerves 4x (wild DISCO)	Voxel Dice	53.29%	45.27%	48.49%	31.80%	20.84%	54.40%	39.33%	41.56%	62.36%
	Centerline Dice	63.12%	57.77%	59.79%	37.19%	27.14%	63.96%	52.19%	40.59%	71.18%
Thy1-EGFP 1x	Voxel Dice	63.97%	65.91%	62.68%	59.27%	56.35%	66.91%	68.05%	50.54%	71.43%
	Centerline Dice	73.10%	69.97%	71.31%	61.93%	68.04%	78.23%	77.18%	43.66%	78.89%

Supplementary Table 3: Evaluation of *Cd68*-eGFP marker segmentation network. We report the average voxel and instance Dice scores, obtained on the test set at the end of training. Best scores are highlighted in bold. Our final model (MouseMapper) was developed by finetuning a foundation model.

	AttentionUnet	nnFormer	UNETR	VNET	nn-Unet	**Mouse Mapper**
Voxel Dice (%)	71.75	75.25	68.25	73.86	75.07	**78.78**
Instance Dice (%)	84.5	84.54	82.97	84.73	85.49	**87.01**

Supplementary Table 4: Transfer learning abilities of the *Cd68*-eGFP segmentation network on new tissue types.

**Organ**	**Instance Dice score**
Stomach	62.58%
Colon	66.55%
Liver	62.77%

Supplementary Table 5: Transfer learning abilities of MouseMapper Immune-Module on CD45 antibody labeled tissues (liver, fat and muscle) and higher resolution 4x *Cd68*-eGFP images.

	CD45_liver	CD45_fat	CD45_muscle	CD68_4x
Voxel Dice	0.64	0.63	0.99	0.83
Instance Dice	0.84	0.74	0.98	0.81

Supplementary Table 6: Benchmarking the Immune-Module against other methods for Cell segmentation. ZS: Zero-shot, out-of-the-box performance of the trained method without finetuning.

Method	Voxel Dice (%)	Instance Dice (%)
CellPose (ZS)	14.29	5.38
AnyStar(ZS)	16.37	39.93
AnyStar-finetuned	42.16	69.38
StarDist(ZS)	1.62	1.20
StarDist-finetuned	54.68	66.97
SCP Nano(ZS)	44.15	53.5
DeepMact(ZS)	2.14	5.31
MouseMapper	78.78	87.01

Supplementary Table 7: Organ segmentation performance of different baselines, per organ. We evaluated state of the art architectures for 3D structure segmentation on a stand-alone test set, comprised of 4 whole body mice (2 HFD, 2 chow). We report the average voxel Dice score (±SD), per organ.

C#	Organ	MouseMapper	AttentionUnet	VNet	nnFormer	SwinUNETR
1	**Spleen**	0.916 ± 0.058	0.894 ± 0.072	0.875 ± 0.099	0.853 ± 0.136	0.857 ± 0.131
2	**Kidney**	0.926 ± 0.102	0.925 ± 0.104	0.891 ± 0.155	0.741 ± 0.33	0.756 ± 0.405
3	**Lungs**	0.973 ± 0.005	0.975 ± 0.004	0.957 ± 0.006	0.949 ± 0.01	0.948 ± 0.02
4	**Heart**	0.976 ± 0.006	0.977 ± 0.005	0.964 ± 0.008	0.955 ± 0.017	0.958 ± 0.022
5	**Brain**	0.987 ± 0.002	0.987 ± 0.002	0.977 ± 0.005	0.976 ± 0.007	0.983 ± 0.004
6	**Gallbladder**	0.781 ± 0.065	0.793 ± 0.06	0.574 ± 0.114	0.761 ± 0.056	0.564 ± 0.136
7	**Adrenal gland**	0.946 ± 0.02	0.931 ± 0.028	0.893 ± 0.051	0.834 ± 0.038	0.775 ± 0.183
8	**Liver**	0.971 ± 0.009	0.971 ± 0.009	0.953 ± 0.023	0.925 ± 0.049	0.945 ± 0.033
9	**Thymus**	0.954 ± 0.019	0.943 ± 0.027	0.934 ± 0.041	0.94 ± 0.019	0.937 ± 0.023
10	**Lymph nodes**	0.937 ± 0.018	0.934 ± 0.018	0.886 ± 0.022	0.889 ± 0.036	0.91 ± 0.025
11	**Stomach**	0.844 ± 0.102	0.818 ± 0.128	0.715 ± 0.28	0.752 ± 0.192	0.658 ± 0.263
12	**Small Intestine**	0.754 ± 0.063	0.752 ± 0.069	0.609 ± 0.13	0.735 ± 0.058	0.674 ± 0.11
13	**Colon**	0.58 ± 0.181	0.576 ± 0.191	0.477 ± 0.225	0.58 ± 0.143	0.472 ± 0.227
14	**Diaphragm**	0.832 ± 0.009	0.818 ± 0.013	0.795 ± 0.028	0.763 ± 0.033	0.775 ± 0.04
15	**Pancreas**	0.623 ± 0.058	0.605 ± 0.063	0.47 ± 0.14	0.563 ± 0.058	0.444 ± 0.133
16	**Abdominal wall**	0.836 ± 0.079	0.836 ± 0.077	0.801 ± 0.062	0.788 ± 0.063	0.825 ± 0.066
17	**Spinal cord**	0.888 ± 0.033	0.888 ± 0.034	0.869 ± 0.018	0.862 ± 0.018	0.866 ± 0.032
18	**Testes**	0.961 ± 0.014	0.962 ± 0.014	0.891 ± 0.102	0.854 ± 0.171	0.899 ± 0.077
19	**Preputial gland**	0.742 ± 0.288	0.699 ± 0.375	0.772 ± 0.217	0.701 ± 0.37	0.712 ± 0.323
20	**Peyer patches**	0.601 ± 0.124	0.579 ± 0.153	0.488 ± 0.23	0.249 ± 0.183	0.512 ± 0.216
21	**Vesicular gland**	0.952 ± 0.012	0.95 ± 0.016	0.919 ± 0.033	0.917 ± 0.042	0.936 ± 0.022
22	**Bladder**	0.671 ± 0.126	0.589 ± 0.213	0.514 ± 0.298	0.541 ± 0.22	0.446 ± 0.195
23	**Submandibular Gland**	0.956 ± 0.018	0.947 ± 0.029	0.883 ± 0.136	0.906 ± 0.052	0.834 ± 0.212
24	**Sublingual Gland**	0.879 ± 0.04	0.881 ± 0.045	0.737 ± 0.272	0.729 ± 0.165	0.772 ± 0.214
25	**Parotid Gland**	0.889 ± 0.016	0.89 ± 0.015	0.866 ± 0.012	0.843 ± 0.009	0.883 ± 0.02
26	**Extraorbital Lacrimal Gland**	0.908 ± 0.026	0.903 ± 0.022	0.863 ± 0.044	0.878 ± 0.026	0.907 ± 0.019
27	**Orbital Lacrimal Gland**	0.856 ± 0.039	0.845 ± 0.032	0.732 ± 0.074	0.764 ± 0.082	0.846 ± 0.042
Average		**0.862 ± 0.055**	0.852 ± 0.065	0.797 ± 0.101	0.795 ± 0.092	0.789 ± 0.114

Supplementary Table 8: Tissue segmentation performance of different architectures for the indicated tissue types. We report the voxel Dice score of 5-fold cross validation result on the final epoch. Best performance is highlighted in bold.

Tissue\ Network architecture	AttentionUnet	NNFormer	UNETR	VNET	3D UNet
Fat	0.904±0.017	0.864±0.038	0.864±0.032	0.152±0.304	**0.908±021**
Muscle	0.958±0.014	0.933±0.013	0.941±0.015	0.157±0.314	**0.961±0.009**
Bone	0.740±0.061	0.669±0.046	0.671±0.059	0.102±0.203	**0.756±0.046**
Bone Marrow	0.881±0.055	0.853±0.037	0.865±0.018	0.129±0.258	**0.887±0.028**
Mean±SD	0.873±0.028	0.832±0.018	0.839±0.020	0.224±0.228	**0.880±0.017**

Supplementary Table 9: Benchmarking the Tissue-Module against other segmentation networks. Model performance was evaluated based on the voxel Dice score. Values of MouseMapper are the same to the values shown in Supplementary Table 7.

#	Organ	AIMOS v1 [1]	Baseline uCT 1 [2]	Baseline uCT 2 [3]	MouseMapper
1	Spleen	0.11	0		0.916
2	Kidney	0.47	0		0.926
3	Lungs	0.35	0.007	0.0008	0.973
4	Heart	0.66	0	0.00014	0.976
5	Brain	0.793			0.987
6	Gallbladder				0.781
7	Adrenal gland				0.946
8	Liver	0.47	0		0.971
9	Thymus				0.954
10	Lymph nodes				0.937
11	Stomach		0		0.844
12	Small intestine		0		0.754
13	Colon		0		0.58
14	Diaphragm				0.832
15	Pancreas				0.623
16	Abdominal wall				0.836
17	Spinal cord			0.022	0.888
18	Testes				0.961
19	Preputial gland				0.742
20	Peyer patches				0.601
21	Vesicular gland				0.952
22	Bladder				0.671
23	Submandibular Gland				0.956
24	Sublingual Gland				0.879
25	Parotid Gland				0.889
26	Extraorbital Lacrimal Gland				0.908
27	Orbital Lacrimal Gland				0.856
T1	Fat				0.908
T2	Muscle				0.961
T3	Bone				0.756
T4	Bone marrow				0.887

Supplementary Table 10: Organs and tissue volumes from *Cd68* and *Uchl1*-eGFP mouse scans were determined by AI-based segmentation and depicted in mm^3.

***Uchl1*-eGFP**	***Uchl1*-eGFP Chow mean**	***Uchl1*-eGFP Chow +/-SEM**	***Uchl1*-eGFP HFD mean**	***Uchl1*-eGFP HFD +/- SEM**	**p-value**
spleen	22.6	1.38	20.4	0.70	0.171739
kidney	131.1	3.02	106.8	5.08	0.006421
lungs	184.0	27.22	132.3	12.56	0.105024
heart	135.2	4.06	123.1	5.27	0.124688
brain	198.3	8.97	218.5	17.85	0.383736
gallbladder	2.2	0.60	2.0	0.17	0.790885
adrenal gl	1.4	0.07	1.4	0.14	0.824754
liver	516.6	11.52	793.8	126.82	0.095990
thymus	9.0	0.77	10.1	1.17	0.496051
lymph nodes	15.9	1.26	21.6	1.11	0.011792
gut	579.6	93.70	394.2	90.03	0.200286
diaphragm	57.6	3.51	60.8	3.96	0.569163
pancreas	105.0	5.82	84.1	14.30	0.258020
abd_wall	500.0	12.24	537.6	34.28	0.382408
spinal_c	49.2	1.51	44.2	3.02	0.214756
testes	45.5	2.14	34.0	2.79	0.016630
prep_gland	34.9	3.88	20.4	3.48	0.027644
peyer_p	2.0	0.34	1.6	0.35	0.519691
vesicular_gl	145.5	17.21	153.3	15.45	0.747480
bladder	18.7	4.13	13.2	2.38	0.266987
submandibular gland	49.9	4.02	48.9	4.18	0.874943
sublingual gland	6.5	0.27	5.2	0.25	0.007023
parotid gland	32.1	3.35	32.5	3.74	0.945720
extraorbital lacrimal gland	10.6	1.68	9.0	1.02	0.416304
orbital lacrimal gland	1.7	0.10	1.4	0.35	0.529407
subcutaneous fat	1749.1	200.99	7005.6	354.68	0.000006
visceral fat	1053.8	129.26	3212.2	245.22	0.000180
muscle	4163.3	175.77	3825.3	116.32	0.139874
bone	249.7	30.68	207.1	36.24	0.415422
bone marrow	243.2	48.28	204.9	52.63	0.616649
***Cd68*-eGFP**	***Cd68*-eGFP Chow mean**	***Cd68*-eGFP Chow +/-SEM**	***Cd68*-eGFP HFD mean**	***Cd68*-eGFP HFD +/- SEM**	**p-value**
spleen	21.47394	2.89	25.37	4.99	0.517605
kidney	131.1079	13.69	132.04	15.94	0.965876
lungs	160.1464	18.84	123.65	10.59	0.129798
heart	140.6311	15.43	143.99	6.79	0.846948
brain	237.1576	6.92	218.32	14.72	0.280154
gallbladder	2.500639	0.66	2.89	0.35	0.620311
adrenal gland	1.497025	0.13	1.55	0.12	0.755659
liver	542.5859	20.69	987.13	28.70	0.000002
thymus	9.344188	0.92	9.49	0.61	0.894933
lymph nodes	13.45823	1.40	19.98	1.53	0.013603
stomach	88.1766	8.78	73.19	8.31	0.250243
gut	364.3868	31.81	256.76	36.66	0.057410
diaphragm	48.74178	1.72	60.46	3.72	0.021047
pancreas	92.39173	16.97	83.28	21.14	0.745479
abd_wall	457.3034	20.17	571.49	61.75	0.116841
spinal_c	53.42271	3.95	42.23	4.08	0.084059
testes	46.8351	4.50	49.17	3.53	0.693607
prep_gland	34.64791	4.11	35.47	6.38	0.916772
peyer_p	1.005661	0.18	1.83	0.38	0.086450
vesicular_gland	120.5856	14.38	156.70	21.60	0.201514
bladder	21.86292	2.54	20.52	3.55	0.765250
submandibular gland	51.69836	3.26	48.27	1.94	0.393072
sublingual gland	6.599308	0.52	5.86	0.44	0.306315
parotid gland	30.56211	1.85	28.19	1.74	0.379096
extraorbital lacrimal gland	12.41727	0.79	10.24	0.58	0.057507
orbital lacrimal gland	1.44953	0.19	1.30	0.09	0.510149
visceral fat	1278.512	182.48	3355.28	118.56	0.000012
bone marrow	249.2223	34.83	237.43	41.77	0.833779
subcutaneous fat	2337.805	335.23	7552.72	241.08	0.000001
bone	188.2028	23.42	191.50	30.69	0.933974
muscle	4058.442	118.91	4108.08	121.80	0.777986

Supplementary Table 11: Characteristics of lean and obese individuals. Characterization of human subjects from which trigeminal ganglia have been isolated post-mortem. Individuals were stratified on the body mass index (BMI) in lean (BMI < 25) or obese (BM I≥ 30). ICD codes indicate cause of death, diseases or medical conditions.

Sex	Age (years)	ICD codes	Weight (kg)	Height (cm)	BMI	Group
female	88.08	N17.9, N18.3, C34.9, I10.9	70	169	24.5	lean
male	83.86	G93.4, F01, N18.3	51	155	21.2	lean
male	83.88	A41, C34	65	173	21.7	lean
female	94.12	N17.9, D63.8, D47.9, I48.2, N18.4, I27.9	53	157	21.5	lean
female	92.07	I24.8, J69.0, I48.0, F03	46	146	21.6	lean
female	72.54	I50.1; I35.0; Z95.2	122	175	39.8	obese
female	94.27	I46.1; I48.9; I10; N18.4	66	147	30.5	obese
female	80.67	K72.1; K75.0; N17.83; I71.1; I11.0	120	169	42.0	obese
female	83.14	I69.4, I67.8	98	153	41.9	obese
