# Supplementary material for: A deep-learning framework reveals whole-body perturbations at cell level
Source: Nature. 2026 May 20;655(8124):1016–26. doi: 10.1038/s41586-026-10535-2 (PMC13391364; doi:10.1038/s41586-026-10535-2)
Supplement: Supplementary file 2 — Reporting Summary [file 41586_2026_10535_MOESM2_ESM.pdf]

nature portfolio

Corresponding author(s): Ali Ertürk

Last updated by author(s): 23/2/26

Reporting Summary

Nature Portfolio wishes to improve the reproducibility of the work that we publish. This form provides structure for consistency and transparency in reporting. For further information on Nature Portfolio policies, see our Editorial Policies and the Editorial Policy Checklist.

Statistics

For all statistical analyses, confirm that the following items are present in the figure legend, table legend, main text, or Methods section.

n/a	Confirmed	
☐	☒	The exact sample size (*n*) for each experimental group/condition, given as a discrete number and unit of measurement
☐	☒	A statement on whether measurements were taken from distinct samples or whether the same sample was measured repeatedly
☐	☒	The statistical test(s) used AND whether they are one- or two-sided<br>*Only common tests should be described solely by name; describe more complex techniques in the Methods section.*
☒	☐	A description of all covariates tested
☐	☒	A description of any assumptions or corrections, such as tests of normality and adjustment for multiple comparisons
☐	☒	A full description of the statistical parameters including central tendency (e.g. means) or other basic estimates (e.g. regression coefficient) AND variation (e.g. standard deviation) or associated estimates of uncertainty (e.g. confidence intervals)
☐	☒	For null hypothesis testing, the test statistic (e.g. *F*, *t*, *r*) with confidence intervals, effect sizes, degrees of freedom and *P* value noted<br>*Give P values as exact values whenever suitable.*
☒	☐	For Bayesian analysis, information on the choice of priors and Markov chain Monte Carlo settings
☒	☐	For hierarchical and complex designs, identification of the appropriate level for tests and full reporting of outcomes
☒	☐	Estimates of effect sizes (e.g. Cohen's *d*, Pearson's *r*), indicating how they were calculated

Our web collection on statistics for biologists contains articles on many of the points above.

Software and code

Policy information about availability of computer code

Data collection	ImspectorPro [v 5.1] was used for collecting light-sheet images. Arivis [v3.0.1 and v3.4] was used for whole-body reconstructions. syGlass (v2.0.0) was used for reference data annotation and visualizations.
Data analysis	cc3d library (Silversmith, W. cc3d: Connected components on multilabel 3D & 2D images. Version 3.2.1. Zenodo https://doi.org/10.5281/zenodo.5719536 (2021)), MONAI (MONAI: Medical Open Network for AI (0.8.1). Zenodo, doi:https://doi.org/10.5281/zenodo.6114127 (2021), DELIVR (Kaltenecker, D. et al.,2024, doi:10.1038/s41592-024-02245-2 (2024)), DIA-NN (Demichev et al, 2020, doi: 10.1038/s41592-019-0638-x), scanpy (v. 1.10.1), anndata (v. 0.8.0), Python 3.8 & 3.10., KNNImputer (n_neighbors=5) from sklearn package (v. 1.2.1), GraphPad Prism (v.9), Microsoft Excel (v2016), spatiomic package (version 0.8.0), DAVID annotation tool (v. Dec. 2021, Knowledgebase v. 2023q4), Our code is available here: https://github.com/erturklab/mouseMapper

For manuscripts utilizing custom algorithms or software that are central to the research but not yet described in published literature, software must be made available to editors and reviewers. We strongly encourage code deposition in a community repository (e.g. GitHub). See the Nature Portfolio guidelines for submitting code & software for further information.

Data

Policy information about availability of data

All manuscripts must include a data availability statement. This statement should provide the following information, where applicable:

- Accession codes, unique identifiers, or web links for publicly available datasets
- A description of any restrictions on data availability
- For clinical datasets or third party data, please ensure that the statement adheres to our policy

MassSpec raw data libraries and outputs from the search engine have been deposited to the ProteomeXchange Consortium via the PRIDE partner repository and made available to the reviewers. Code will be made available here: https://github.com/erturklab/mouseMapper
Whole-body mouse atlases were made available online to scroll through. Links can be found at https://discotechnologies.org/MouseMapper/

Human research participants

Policy information about studies involving human research participants and Sex and Gender in Research.

Reporting on sex and gender	Sex of the human sample donors is indicated in the manuscript
Population characteristics	The average age of lean human sample donors was 88.4 years and 82.6 years for obese human sample donors. Details for each Donor is indicated in Supplementary Table 11
Recruitment	For this study, only donors within two BMI categories were included: BMI < 25 kg/m², classified as lean and BMI > 30 kg/m², classified as obese, following standard clinical thresholds. No additional selection criteria regarding sex, age, comorbidities, or cause of death were applied beyond the requirement. Because samples were obtained opportunistically from available donors, self-selection bias is minimal, as donors did not actively self-enroll. However, availability bias may be present due to demographic patterns of tissue donation and medical autopsy, which could affect the distribution of age and sex.
Ethics oversight	Institute of Anatomy, University of Leipzig, Germany. Ethical approval number 129/21-ck, Medizinische Fakultät Ethik-Kommission

Note that full information on the approval of the study protocol must also be provided in the manuscript.

Field-specific reporting

Please select the one below that is the best fit for your research. If you are not sure, read the appropriate sections before making your selection.

☒ Life sciences ☐ Behavioural & social sciences ☐ Ecological, evolutionary & environmental sciences

For a reference copy of the document with all sections, see nature.com/documents/nr-reporting-summary-flat.pdf

Life sciences study design

All studies must disclose on these points even when the disclosure is negative.

Sample size	The sample size of 19 mice for whole-body analysis (4 chow UCHL1-EGFP, 5 HFD UCHL1-EGFP, 5 chow CD68-EGFP and 5 HFD UCHL1-EGFP) was chosen due to the feasibility of conducting light-sheet imaging, 3D reconstructions and AI-analysis of large-scale data.
Data exclusions	No animals were excluded from the study.
Replication	The whole-body vDISCO labeleig protocol, tissue-clearing, imaging, segmentation, mapping and quantification procedures were successfully performed on 19 mice for this project.
Randomization	Animals were randomly assigned to chow or HFD groups.
Blinding	No blinding was done because knowledge of experimental conditions was required during animal handling and data collection.

Reporting for specific materials, systems and methods

We require information from authors about some types of materials, experimental systems and methods used in many studies. Here, indicate whether each material, system or method listed is relevant to your study. If you are not sure if a list item applies to your research, read the appropriate section before selecting a response.

Materials & experimental systems

n/a	Involved in the study
☐	☒ Antibodies
☒	☐ Eukaryotic cell lines
☒	☐ Palaeontology and archaeology
☐	☒ Animals and other organisms
☒	☐ Clinical data
☒	☐ Dual use research of concern

Methods

n/a	Involved in the study
☒	☐ ChIP-seq
☒	☐ Flow cytometry
☒	☐ MRI-based neuroimaging

Antibodies

Antibodies used	Atto647N-conjugated anti-GFP nanobooster (Chromotek Cat.# gba647n-100; RRID:AB_2629215), SEPTIN7 (Proteintech, #13818-1-AP, 1:1000), SERPINA3 (Proteintech #16382-1-AP, 1:1000), p-ERK (Cell Signaling, Phospho-p44/42 MAPK (Thr202/Tyr204) #9101, 1:1000), ERK (p44/42 MAPK, #9102, 1:1000), Vinculin (EPR8185, Abcam, #ab129002, 1:10000), Goat-Anti Rabbit IgG (Bio-Rad, #1721019), PGP9.5 (Proteintech, 14730-1-AP1, 26µl in 200 ml solution), anti-CGRP (ab36001, Abcam, 26 µl in 200 ml solution)
Validation	Statements about the anti-GFP nanobooster can be found on the website of the mannfacurer (https://www.ptglab.com/products/GFP-Booster-ATTO647N-gba647n.htm#product-information): The nanobodies are validated by a genetic approach. They are tested on cell lines that express and do not express GFP. TThe manufacturers validated the Western blotting antibodies and information can be found on the respective Website of the manufacturer: https://www.ptglab.com/de/products/SERPINA1-Antibody-16382-1-AP.htm , https://www.ptglab.com/de/products/SEPT7-Antibody-13818-1-AP.htm , https://www.cellsignal.com/products/primary-antibodies/phospho-p44-42-mapk-erk1-2-thr202-tyr204-antibody/9101?srsltid=AfmBOoqnwT3mMA5lJkHsluTWp6PLIYmdR0oM7n4EPgacdCnb3E_J9P1H , https://www.cellsignal.com/products/primary-antibodies/p44-42-mapk-erk1-2-antibody/9102?srsltid=AfmBOorMCQDqxJRBCnDa6wnIiIUBTarrwTVppcRcBNvDHNhMk8qnbapo. PGP9.5 was knockdown/KO validated (https://www.ptglab.com/de/products/UCHL1-Antibody-14730-1-AP.htm?srsltid=AfmBOoooWGX1eUx5DHRss-xB7jAcwisHgJdJHRu4Bwev5mRdI9qB2IMF); Validation about CGRP can be found here: https://www.abcam.com/en-us/products/primary-antibodies/cgrp-antibody-ab36001

Animals and other research organisms

Policy information about studies involving animals; ARRIVE guidelines recommended for reporting animal research, and Sex and Gender in Research

Laboratory animals	Male UCHL1-EGFP and CD68–EGFP mice (on a C57BL/6J background) and C57BL/6J wildtype mice were put on HFD at an age of 8 weeks for 16-18 weeks.
Wild animals	We did not use wild animals.
Reporting on sex	We used only used male mice in our study to keep experimental conditions maintain consistency in experimental conditions and minimize variability.
Field-collected samples	The study did not involve samples collected from the field.
Ethics oversight	Animal experimentation was performed in accordance with the European Union directives and the German animal welfare act (Tierschutzgesetz). They have been approved the state ethics committee and the government of Upper Bavaria.

Note that full information on the approval of the study protocol must also be provided in the manuscript.
